# MALDI Mass Spectrometry Imaging in Alzheimer’s Disease Lipidomics: Matrix Selection, Spatial Lipid Pathology and Emerging Analytical Strategies

**DOI:** 10.3390/ijms27167074

**Published:** 2026-08-07

**Authors:** David Aebisher, Anna Krzysztofińska, Barbara Smolak, Patrycja Bernat, Wiktoria Czajka, Magdalena Kowal, Sylwia Krasoń, Klaudia Dynarowicz, Wiesław Guz, Dorota Bartusik-Aebisher

**Affiliations:** 1 Department of Photomedicine and Physical Chemistry, Faculty of Medicine, University of Rzeszów, 35-310 Rzeszów, Poland; 2 Medical Science Club, Faculty of Medicine, University of Rzeszów, 35-310 Rzeszów, Poland; ak121978@stud.ur.edu.pl (A.K.); pb117718@stud.ur.edu.pl (P.B.); wc121927@stud.ur.edu.pl (W.C.); mk122013@stud.ur.edu.pl (M.K.); sk098764@stud.ur.edu.pl (S.K.); 3 Department of Diagnostic Imaging and Nuclear Medicine, Faculty of Medicine, University of Rzeszów, 35-310 Rzeszów, Poland; bsmolak@ur.edu.pl (B.S.); wguz@ur.edu.pl (W.G.); 4 Department of Biochemistry and General Chemistry, Faculty of Medicine, University of Rzeszów, 35-310 Rzeszów, Poland; kdynarowicz@ur.edu.pl

**Keywords:** Alzheimer’s disease, MALDI mass spectrometry imaging, spatial lipidomics, amyloid plaques, gangliosides, sulfatides, matrix selection, ion mobility, on-tissue derivatization, molecular neuropathology

## Abstract

Alzheimer’s disease (AD) involves not only amyloid-β and tau pathology but also extensive disturbances in lipid metabolism, membrane organization, neuroinflammatory signaling, and tissue homeostasis. Conventional lipidomics has identified changes in phospholipids, sphingolipids, sulfatides, ceramides, gangliosides, and cholesterol-related pathways, but tissue homogenization removes their anatomical context. The aim of this review is to critically assess how matrix selection, sample preparation, ionization polarity, and emerging analytical strategies influence the detection and interpretation of spatial lipid alterations specifically associated with AD neuropathology. Current evidence shows that AD-related lipid remodeling is region- and lesion-specific, with recurrent findings including ganglioside accumulation, sulfatide depletion, ceramide-related alterations, phospholipid remodeling, lysosomal lipid changes, and disturbed cholesterol homeostasis within or around amyloid plaques. Matrix chemistry strongly influences lipid-class coverage, ionization efficiency, spectral background, adduct formation, spatial resolution, and biological interpretation. Matrix-Assisted Laser Desorption/Ionization with Laser-Induced Post-Ionization (MALDI-2), ion mobility, reactive matrices, on-tissue derivatization, structural lipidomics, single-cell imaging, and spatial multiomics are expanding molecular coverage and annotation confidence. However, broader translation requires standardized workflows, structurally validated assignments, quantitative quality control, larger human cohorts, and improved interlaboratory reproducibility. Collectively, the available evidence indicates that the principal value of Matrix-Assisted Laser Desorption/Ionization Mass Spectrometry Imaging (MALDI-MSI) in AD lies not merely in detecting altered lipid abundance, but in resolving lesion-specific lipid microenvironments whose interpretation depends directly on matrix chemistry, spatial resolution, and structural validation.

## 1. Introduction

Alzheimer’s disease (AD) is a progressive neurodegenerative disorder characterized by cognitive decline, synaptic and neuronal loss, extracellular amyloid-β (Aβ) deposition, intracellular accumulation of pathologically modified tau, and complex glial and inflammatory responses. Although amyloid and tau remain central components of AD neuropathology, they do not fully explain the molecular heterogeneity, regional vulnerability, or variable clinical progression of the disease. Increasing evidence therefore supports a broader model in which metabolic dysfunction, particularly disturbed lipid homeostasis, participates in disease initiation and progression rather than representing only a secondary consequence of neurodegeneration [1,2].

Lipids are essential for the structural and functional organization of the central nervous system. They form neuronal and glial membranes, maintain myelin integrity, regulate membrane curvature and fluidity, support synaptic vesicle cycling, and provide precursors for bioactive signaling molecules. Human brain lipidomic studies have demonstrated that AD is associated with alterations extending across glycerophospholipids, sphingolipids, neutral lipids, fatty acids, and lysophospholipids, although the direction and magnitude of these changes depend on the brain region, disease stage, cellular composition, and analytical workflow [3,4,5].

**Figure 1:**
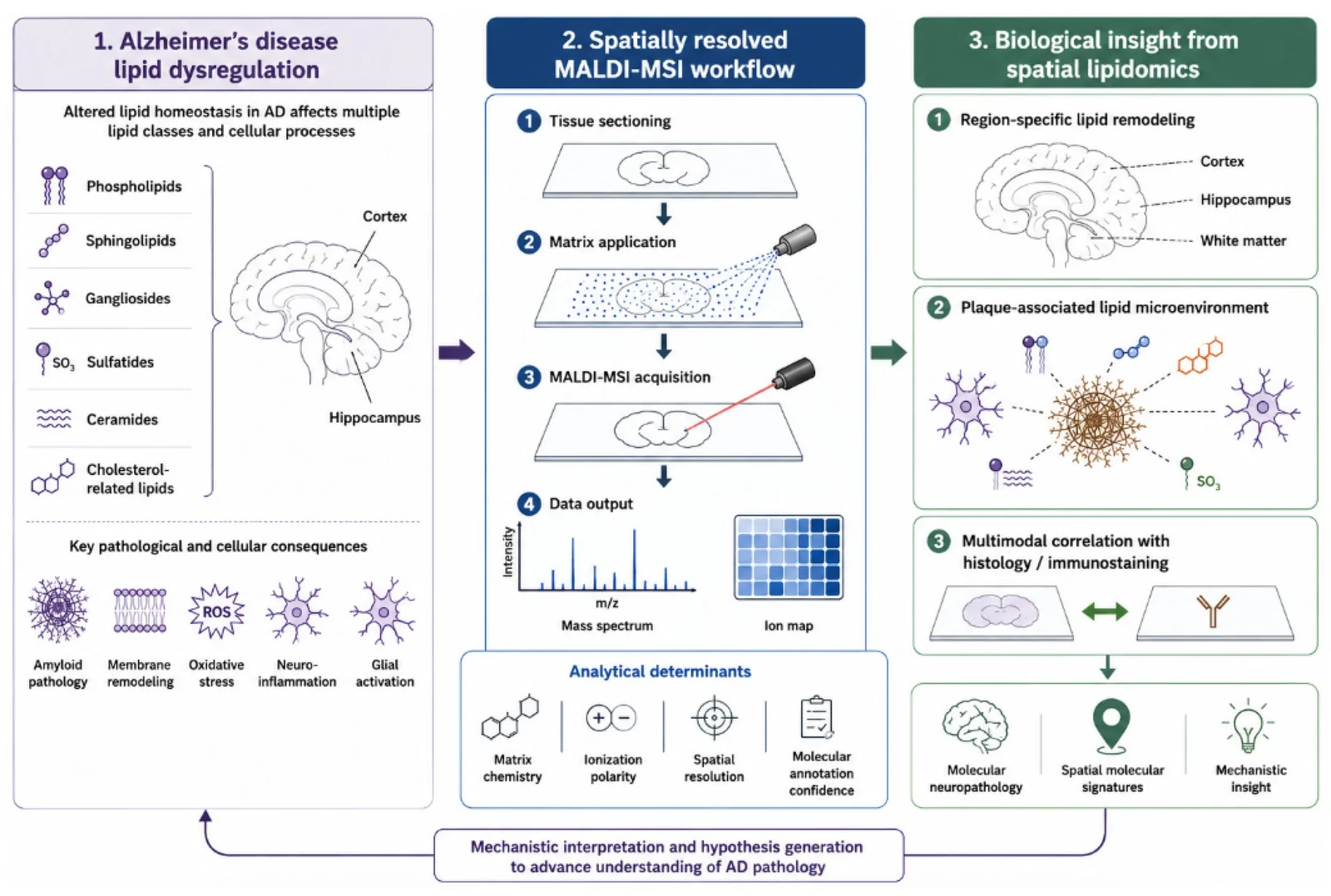
Alzheimer’s disease-specific framework linking neuropathological processes with spatial lipid remodeling detected by MALDI-MSI.

The biological relevance of lipid dysregulation extends beyond changes in total lipid abundance. Phosphatidylcholines, phosphatidylethanolamines, phosphatidylinositols, lysophospholipids, sphingomyelins, ceramides, sulfatides, gangliosides, and cholesterol-related species participate in membrane organization, protein trafficking, signal transduction, and cellular stress responses (Figure 1). Their altered metabolism may influence amyloid precursor protein processing, Aβ production and aggregation, tau modification, oxidative damage, myelin disruption, and synaptic instability [1,6,7].

Lipid metabolism is also closely connected with the genetic and immune architecture of AD. Apolipoprotein E regulates cholesterol and phospholipid transport among astrocytes, neurons, and microglia, while the APOE ε4 allele modifies lipid handling, membrane composition, cellular bioenergetics, and inflammatory responses. In parallel, prolonged microglial and astrocytic activation may impair lipid clearance, promote lipid-droplet accumulation, and reinforce chronic neuroinflammation, creating a bidirectional interaction between lipid dyshomeostasis and immune dysfunction. These interactions indicate that lipid abnormalities in AD should be interpreted in relation to amyloid pathology, tau-associated processes, APOE-dependent lipid transport, glial activation, and the anatomical compartments in which these mechanisms converge [2,8].

An important limitation of the available evidence is that lipid abnormalities are not uniform throughout the brain or across disease progression. A systematic evaluation of mouse-model lipidomics revealed substantial variation associated with model type, age, sex, sampled brain region, tissue collection, lipid extraction, and analytical platform [6]. More recent spatial–temporal lipidomic analysis has further shown that lipid species may change in an age- and region-dependent manner during AD progression, indicating that bulk measurements can obscure molecular alterations restricted to vulnerable anatomical structures or discrete pathological niches [9].

Conventional liquid chromatography–mass spectrometry and related extraction-based lipidomic workflows provide high molecular coverage, chromatographic separation, and valuable quantitative information. However, homogenization averages signals from neurons, astrocytes, microglia, oligodendrocytes, vascular elements, myelin, extracellular deposits, and unaffected tissue within the same sample. Consequently, a statistically significant change in a tissue extract cannot reveal whether the responsible lipid is associated with a specific anatomical region, cell-rich compartment, myelinated tract, vascular structure, or amyloid plaque microenvironment [10,11].

Matrix-assisted laser desorption/ionization mass spectrometry imaging (MALDI-MSI) addresses this limitation by detecting molecular ions directly from tissue sections while retaining their spatial coordinates (Figure 1). Early applications in transgenic AD models demonstrated that MALDI-MSI can localize amyloid-related molecular changes and delineate plaque-associated sphingolipid distributions without requiring analyte-specific labeling [12,13]. These studies established that lipid remodeling in AD is spatially organized and that molecular signals associated with plaques may differ from those in adjacent tissue.

Subsequent multimodal approaches have expanded this concept by integrating lipid imaging with Aβ peptide detection, histology, fluorescence microscopy, or immunohistochemical markers. Such workflows have revealed relationships among plaque morphology, ganglioside accumulation, sphingolipid remodeling, and the surrounding tissue response at increasingly refined spatial scales [14,15,16]. Importantly, these findings support the interpretation of amyloid plaques not as chemically uniform deposits, but as heterogeneous molecular microenvironments whose cores, peripheral zones, and surrounding tissue may contain distinct lipid signatures. Accordingly, the principal biological contribution of MALDI-MSI is its ability to determine where lipid remodeling occurs in relation to defined neuropathological structures, rather than merely whether a lipid is increased or decreased in homogenized tissue (Figure 1).

Recent reviews have summarized the broader use of mass spectrometry imaging in AD and other neurological disorders, as well as the emerging contribution of spatial neurolipidomics to neuropathological research [11,17,18]. Nevertheless, a focused synthesis is still required to connect matrix chemistry and sample preparation with the lipid classes detected, the spatial patterns reported in AD tissue, and the biological conclusions derived from those images. This methodological–biological relationship is particularly important because apparent differences among studies may arise not only from disease biology, but also from matrix selectivity, ionization polarity, spatial resolution, tissue handling, spectral interference, and annotation strategy [17,18,19,20].

Matrix selection is therefore not a peripheral preparative choice but a central determinant of the detectable tissue lipidome. Matrix structure, solvent composition, deposition method, crystal dimensions, spectral background, analyte extraction, adduct formation, and ionization polarity influence both molecular coverage and spatial fidelity. Since no single matrix provides equivalent sensitivity for all lipid classes, results obtained with one matrix–polarity combination should not be interpreted as a comprehensive representation of AD-associated lipid remodeling [19,20].

The schematic illustrates the interactions among amyloid-β plaques, tau-associated pathology, APOE ε4-dependent lipid transport, glial activation, myelin alterations, and region-specific neuronal vulnerability. Plaque cores, peri-plaque zones, and surrounding parenchyma are presented as distinct but interacting molecular compartments. The detection and interpretation of gangliosides, sulfatides, ceramides, phospholipids, and cholesterol-related species depend on matrix chemistry, ionization polarity, spatial resolution, and the level of molecular validation.

This review critically evaluates the application of MALDI-MSI to AD lipidomics, with particular emphasis on matrix selection, tissue preparation, matrix deposition, spatial lipid pathology, and the interpretation of plaque-associated molecular patterns. It further examines analytical limitations—including ion suppression, adduct formation, isomeric and isobaric overlap, incomplete molecular annotation, and limited quantitative comparability—and discusses emerging approaches such as MALDI-2, ion mobility separation, multimodal imaging, and on-tissue chemical derivatization. The review specifically examines how analytical choices determine the visibility and interpretation of AD-associated lipid patterns and how spatial evidence can be integrated into a more mechanistic understanding of plaque-associated and region-dependent lipid pathology [17,18,19,20].

## 2. Principles of MALDI and MALDI-MSI in Lipidomics

### 2.1. MALDI Ionization Principles

Matrix-assisted laser desorption/ionization mass spectrometry (MALDI-MS) is a soft-ionization technique that enables the detection of a broad range of biomolecules, including structurally diverse lipid species. In MALDI analysis, the matrix absorbs laser radiation and facilitates analyte desorption and ion formation before mass-to-charge separation [10,19,21].

The matrix is not an analytically passive component. Its chemical structure and physical properties influence analyte extraction, co-crystallization, ion formation, adduct generation, fragmentation, and the stability of the detected species. Matrix chemistry, analyte structure, laser fluence, crystal morphology, tissue salt content, and gas-phase reactions jointly determine which molecular species are ultimately detected [19,20,21].

This dependence is especially important in lipidomics because lipid classes differ markedly in intrinsic charge, acidity, proton affinity, polarity, surface activity, and susceptibility to fragmentation. The recorded spectrum consequently reflects both the tissue lipid composition and the analytical selectivity of the applied matrix, polarity, and acquisition conditions. The absence of a lipid signal cannot therefore be interpreted automatically as evidence that the corresponding molecule is absent from the tissue. In AD studies, this limitation is particularly relevant when comparing the detectability of plaque-associated gangliosides, sulfatides, ceramide-related species, phospholipids, and cholesterol-related lipids across different analytical workflows [10,19,20].

### 2.2. MALDI-MSI Image Generation and Analytical Workflow

MALDI mass spectrometry imaging extends conventional MALDI-MS by acquiring a complete mass spectrum at each coordinate of a predefined raster across a tissue section. Each measurement coordinate constitutes one image pixel, while the intensity of a selected *m*/*z* feature can be reconstructed across all sampled positions to generate a two-dimensional ion image. This approach preserves the anatomical origin of molecular signals and permits direct comparison of lipid distributions with histological structures. In AD tissue, this spatial information enables lipid signals to be related to cortical and hippocampal regions, white matter, vascular structures, and individual amyloid-plaque microenvironments [17,21].

A typical MALDI-MSI workflow includes tissue collection and preservation, sectioning, mounting on a conductive substrate, optional pretreatment, matrix deposition, raster-based spectral acquisition, data preprocessing, peak detection, image reconstruction, molecular annotation, and histological co-registration. Because each stage can alter molecular coverage and spatial fidelity, methodological differences must be considered when comparing lipid distributions reported in AD models and human tissue [17,21].

An ion image represents the spatial distribution of a detected *m*/*z* feature, but it does not necessarily represent one uniquely identified lipid molecule. A single peak may correspond to a protonated or deprotonated lipid, an alkali-metal adduct, an isotope, an in-source fragment, a matrix-related ion, or several unresolved isobaric species. Reporting terminology should therefore distinguish among an unassigned *m*/*z* feature, a putatively annotated lipid, and a structurally confirmed molecular species [10,17,21].

### 2.3. Lipid-Class-Dependent Ionization and Adduct Formation

Ionization polarity is one of the principal determinants of lipid-class coverage in MALDI-MSI. In positive-ion mode, phosphatidylcholines and sphingomyelins are commonly detected with high sensitivity because their phosphocholine headgroup facilitates stable positive-ion formation. Depending on the experimental conditions, these lipids may appear as protonated, sodiated, or potassiated ions. Neutral lipids, including triacylglycerols and cholesterol esters, are often detected predominantly through ammonium or alkali-metal adduct formation [10,19].

Negative-ion mode generally provides improved access to acidic phospholipids and glycosphingolipids. Phosphatidylinositols, phosphatidylserines, phosphatidylglycerols, phosphatidic acids, sulfatides, and gangliosides are frequently observed as deprotonated ions. Phosphatidylethanolamines and selected ceramide-related species may be detected in either polarity, although their response depends on molecular structure, matrix chemistry, tissue composition, and instrumental conditions [10,17,19].

Positive- and negative-ion measurements are therefore complementary rather than interchangeable. A single-polarity experiment may preferentially represent efficiently ionized lipid classes while underestimating chemically less favorable species. Sequential dual-polarity imaging from the same tissue section can broaden lipid coverage and reduce uncertainty caused by anatomical differences between adjacent sections. This complementarity is directly relevant to AD because positive-ion acquisition favors abundant phosphocholine-containing membrane lipids, whereas negative-ion acquisition provides greater access to plaque-associated gangliosides, myelin-related sulfatides, phosphatidylinositols, and other acidic lipid classes [22].

The performance of dual-polarity imaging is also strongly dependent on matrix design. Recently developed aminated cinnamic acid analogues have demonstrated that a single matrix system can support high-spatial-resolution detection of polar lipids in both ionization modes. Such approaches reduce the need for repeated matrix application and facilitate direct comparison of complementary positive- and negative-ion lipid distributions [23].

Adduct formation can improve the detectability of weakly ionizing lipids, but it also increases spectral complexity. Local differences in sodium and potassium abundance may redistribute the signal of one lipid among several adduct forms. Conversely, multiple peaks may be interpreted incorrectly as separate metabolites when they represent protonated, sodiated, or potassiated forms of the same molecule. Annotation should therefore consider expected mass differences, isotope patterns, co-localized spatial distributions, and fragmentation behavior [10,20,21].

Ion suppression presents an additional limitation because analytes within the same pixel compete during extraction, desorption, and ionization. Highly abundant phosphatidylcholines or sphingomyelins may suppress lower-abundance acidic, oxidized, or neutral lipid species. Ion intensity therefore cannot be treated as a direct measure of tissue concentration without appropriate validation. Consequently, apparent differences between plaque-rich and plaque-free regions may reflect both true lipid remodeling and local differences in ionization efficiency [10,20,21].

### 2.4. Spatial Resolution, Mass Resolution, and Sensitivity

Spatial resolution in MALDI-MSI is frequently reported as the raster step or nominal pixel dimension. However, effective lateral resolution is additionally determined by the laser-ablation diameter, beam profile, matrix crystal dimensions, tissue thickness, analyte migration during matrix deposition, stage accuracy, and overlap between adjacent ablation areas. A nominally small pixel size does not therefore guarantee an equivalent independent spatial resolution [17,21].

Oversampling involves moving the sample stage at intervals smaller than the laser-ablation diameter. MALDI-2 post-ionization can partly compensate for the loss of ion yield associated with smaller sampled areas and has enabled high-resolution metabolite and lipid imaging approaching the cellular scale [24].

Increasing spatial resolution creates an inherent sensitivity trade-off. As the sampled tissue area becomes smaller, fewer analyte molecules contribute to each spectrum. High-resolution experiments may consequently produce lower signal-to-noise ratios, reduced molecular coverage, longer acquisition times, and substantially larger datasets. The selected pixel dimension should therefore be determined by the biological question and the size of the target structure rather than by the pursuit of the smallest technically achievable raster step. For AD studies, regional mapping, plaque-level imaging, and cellular-scale analysis require different compromises between spatial resolution and molecular sensitivity [17,21,22,24].

Mass resolving power and mass accuracy address different aspects of spectral interpretation. High resolving power improves separation of ions with closely spaced *m*/*z* values, whereas high mass accuracy narrows the range of plausible elemental compositions. Neither parameter is sufficient to distinguish lipid molecules that share the same elemental formula but differ in fatty-acyl arrangement, sn-position, double-bond position, double-bond geometry, or sphingoid-base structure [17,21].

### 2.5. Molecular Annotation and Interpretative Limitations

Accurate precursor mass usually only supports a putative lipid assignment. At the MS1 level, an ion may often be matched to a lipid class and sum composition, but the identities and positions of individual fatty-acyl chains remain unresolved. Tandem mass spectrometry can provide information about headgroups and fatty-acyl composition, although the quality of structural evidence depends on precursor isolation, ion abundance, fragmentation efficiency, polarity, and adduct type [10,17,21].

Isomerism constitutes a major limitation of conventional lipid MSI. Lipids with identical nominal and exact masses may differ in sn-position, double-bond position, cis/trans geometry, or sphingoid-base composition. These structural variants may have distinct biological functions while remaining indistinguishable in routine MS1 images. Reactive matrices and photochemical derivatization strategies have begun to address this limitation. Light-reactive norharmane derivatization, for example, can generate diagnostic products that support localization of carbon–carbon double bonds in unsaturated lipids [25].

Other approaches, including ion mobility separation, ozone-induced dissociation, ultraviolet photodissociation, Paternò–Büchi reactions, and on-tissue chemical derivatization, can provide additional structural specificity. Nevertheless, no single method resolves every type of lipid isomer, and assignments should remain appropriately qualified unless supported by standards, tandem MS, orthogonal LC–MS/MS, or complementary structural evidence [17,21,25].

Although MALDI is classified as a soft-ionization method, in-source fragmentation may occur when laser fluence, matrix chemistry, source conditions, or ionization polarity are not adequately optimized. Phospholipids may undergo headgroup losses or fatty-acyl cleavage, while gangliosides, sulfatides, plasmalogens, and oxidized lipids contain labile structural elements that may fragment readily. Such fragment ions can be mistaken for endogenous tissue molecules, particularly when their spatial distributions resemble those of their precursors. This is especially important when interpreting plaque-associated ganglioside or sulfatide signals, because fragmentation may generate ions resembling structurally simpler endogenous species [26].

Fragmentation-related misassignment may also distort statistical analyses. Precursor ions, fragments, isotopes, and alternative adducts can be treated as independent variables, artificially increasing the apparent number of disease-associated molecular features. Robust annotation workflows should therefore include isotope recognition, adduct grouping, evaluation of known fragmentation pathways, mass recalibration, and targeted confirmation by tandem MS or orthogonal lipidomics [17,21,26].

MALDI-MSI data are predominantly relative or semi-quantitative. Signal intensity is influenced not only by analyte abundance but also by matrix incorporation, local extraction efficiency, ion suppression, adduct competition, laser–sample interaction, and detector response. Comparisons among anatomical regions, experimental groups, or analytical batches therefore require standardized sample preparation, quality-control sections, consistent normalization, internal standards where feasible, and transparent reporting of analytical variability [20,21].

The principal analytical dimensions affecting lipid MALDI-MSI interpretation are summarized in Table 1. Together, they demonstrate that spatial localization, molecular identification, and signal intensity represent distinct analytical dimensions and should not be treated as equivalent evidence. In AD research, these dimensions determine whether an observed plaque- or region-associated signal can be interpreted as a localized, structurally supported, and biologically meaningful lipid alteration.

Overall, MALDI-MSI should be regarded as an integrated spatial analytical workflow rather than as an autonomous method for complete lipid identification or absolute quantification. Its main strength is the preservation of molecular–anatomical relationships, whereas the reliability of the resulting biological conclusions depends on coordinated optimization of tissue preparation, matrix chemistry, polarity, spatial sampling, mass analysis, molecular validation, and histological interpretation. These dependencies are particularly important in AD research, where relevant lipid changes may be confined to small plaque-associated, cellular, vascular, or myelin-rich microenvironments [17,20,21].

## 3. Sample Preparation and Matrix Deposition Strategies for Lipid MALDI-MSI

Sample preparation is an integral component of lipid MALDI-MSI rather than a preliminary step preceding instrumental analysis. Tissue collection, stabilization, sectioning, mounting, drying, pretreatment, and matrix deposition jointly determine lipid preservation, extraction efficiency, ion formation, and spatial fidelity. These variables are especially important in Alzheimer’s disease (AD), where biologically relevant lipid alterations may be restricted to cortical layers, myelinated tracts, vascular structures, individual amyloid plaques, or narrow plaque-associated microenvironments [17,20,21].

### 3.1. Tissue Collection, Freezing, and Storage

Fresh-frozen tissue is generally preferred for untargeted lipid MALDI-MSI because it avoids formalin-induced chemical modification and the extensive solvent exposure associated with paraffin processing. A sequential workflow developed for fresh-frozen rodent brain demonstrated that the same tissue section can support dual-polarity lipid imaging followed by N-glycan and peptide analysis, provided that tissue handling and the order of analytical procedures are carefully controlled [27].

Rapid stabilization is required because brain tissue contains high concentrations of membrane phospholipids, sphingolipids, cholesterol, and polyunsaturated fatty acids, as well as enzymes capable of modifying these molecules after tissue collection. Delayed cooling may permit phospholipid hydrolysis, lysophospholipid generation, oxidation, and changes in the balance between free and membrane-associated fatty acids. The interval between tissue collection and freezing should therefore be minimized and standardized across all experimental groups [17,27].

Freezing conditions must balance rapid metabolic arrest with preservation of tissue morphology. The selected rapid-freezing procedure should preserve tissue morphology and be standardized for the specimen size and brain region under investigation [17,21,27].

Tissue preservation methods can modify the lipid profile independently of biological condition. Comparative mass spectrometry imaging of differently prepared mouse brain specimens demonstrated that fresh-frozen and chemically preserved tissues differ in the number, intensity, and spatial distribution of detectable lipid-related features. Fixation and cryoprotection may improve mechanical stability but can reduce lipid coverage, modify membrane accessibility, and alter ion-adduct formation [28].

Frozen specimens should be maintained in sealed containers at a stable low temperature, commonly −80 °C, and repeated freeze–thaw cycles should be avoided. Storage duration, temperature history, and the number of thawing events should therefore be documented as preanalytical metadata [27,28,29].

Tissues should remain frozen until cryosectioning, and the time between sectioning, drying, matrix application, and instrumental acquisition should remain consistent across the analytical cohort [29].

Human post-mortem brain tissue introduces additional sources of variation, including post-mortem interval, agonal state, tissue pH, dissection procedure, anatomical sampling, storage history, and previous thawing. These factors may influence lipid oxidation and hydrolysis independently of AD pathology. Case–control studies should therefore match them whenever possible and report them together with age, sex, brain region, neuropathological diagnosis, and disease stage [11,17].

### 3.2. Cryosectioning, Tissue Thickness, Embedding, and Conductive Substrates

Cryosectioning should produce flat, intact tissue sections while preserving molecular composition and anatomical orientation. Brain tissue is commonly sectioned at approximately 8–14 μm for lipid MALDI-MSI, although the optimal thickness depends on tissue rigidity, instrument geometry, laser penetration, matrix application, and the required balance between sensitivity and spatial resolution [21,27].

A systematic investigation of section thickness showed that the optimal value is platform-dependent and that conventional vacuum MALDI and atmospheric-pressure configurations may not provide equivalent responses from the same tissue thickness. Section thickness should therefore be selected and reported consistently because it influences both spatial definition and analyte yield [30].

Sectioning temperature must be adapted to tissue composition. Sectioning conditions should be standardized to avoid deformation of lipid-rich white matter and variable section integrity [27,29,30].

Sections used for group comparisons should be obtained from comparable anatomical levels. Even small differences in sectioning depth may alter the proportions of cortical layers, hippocampal subfields, white matter, vasculature, and plaque-rich regions. Anatomical variation can consequently produce apparent lipid differences that are unrelated to disease status [11,17].

Embedding media require particular caution because compounds used to support cryosectioning may interfere with MALDI analysis. Optimal cutting temperature compounds contain polymers and additives that can generate background ions, contaminate the ion source, alter matrix crystallization, and suppress endogenous signals. Direct contact between OCT and the tissue surface intended for analysis should therefore be avoided [17,21].

Carboxymethyl cellulose and gelatin-based systems have been investigated as alternatives for small or mechanically unstable specimens. Sodium carboxymethyl cellulose provided sufficient mechanical support for sectioning multicellular spheroids while reducing some of the analytical problems associated with OCT exposure. Nevertheless, any embedding material should be tested in blank controls because its spectral compatibility depends on composition, ionization polarity, and mass range [31].

Embedding-medium blanks should be included to distinguish polymer- or additive-derived signals from endogenous lipid features [21,31].

Tissue sections are commonly thaw-mounted on indium tin oxide-coated glass slides because ITO provides electrical conductivity while remaining compatible with optical microscopy and subsequent histological co-registration.

Section adhesion should be inspected before matrix deposition. Tissue folds, tears, air pockets, partial detachment, and uneven contact with the slide may produce local signal loss that resembles biological heterogeneity. Optical images acquired immediately after mounting and again after matrix application provide an essential record of section integrity [17,21].

### 3.3. Drying, Tissue Pretreatment, Washing, and Desalting

Mounted sections are usually dried under controlled low-humidity conditions before matrix application. Vacuum desiccation limits condensation and provides a reproducible tissue surface for matrix deposition. In the sequential rodent-brain workflow, vacuum equilibration was incorporated before application of norharmane for dual-polarity lipid imaging [27].

Routine washing is not universally appropriate for lipid MALDI-MSI. Lipid classes differ substantially in polarity, membrane association, solubility, and susceptibility to extraction. A solvent treatment that enhances the detection of one class may remove another, change local ion suppression, modify salt distributions, or induce lateral migration [17,20].

A basic hexane pretreatment was shown to improve MALDI imaging of polar and isotope-labelled metabolites by removing interfering tissue components. Analysis of the recovered wash solution, however, demonstrated simultaneous extraction of several lipid classes. This illustrates that improved detection of retained analytes may be achieved at the cost of incomplete representation of the native lipidome [32].

Systematic comparison of wash solvents has confirmed that aqueous and organic treatments produce markedly different changes in molecular-class retention and ion suppression. Washing conditions should therefore be selected according to the analytical target rather than transferred directly from protein or metabolite MSI workflows. In AD studies, pretreatment must preserve plaque-associated and regional lipid distributions rather than maximize detection of an unrelated molecular class [33].

Desalting may simplify spectra by reducing alkali-metal adduct formation, but it can also remove analytically useful information. Many phospholipids and neutral lipids are detected as sodium or potassium adducts, and regional cation differences may influence their observed distributions. The effects of washing should consequently be evaluated separately in positive- and negative-ion modes [10,20,33].

Pretreatment validation should include spatial as well as spectral criteria. Appropriate endpoints include preservation of tissue boundaries, retention of grey–white matter contrast, absence of signal outside the section, reproducibility across serial sections, and analysis of compounds recovered in the wash solution. Orthogonal LC–MS analysis of wash extracts can reveal molecular losses that are not evident from the final ion images [20,32,33].

Sequential multimodal workflows require a predefined analytical order. Lipid MSI is usually performed before delipidation, enzymatic digestion, antigen retrieval, or aqueous histological staining because these procedures can substantially modify the lipid profile. Subsequent matrix removal and downstream analysis are feasible, but each transition step must be evaluated for tissue integrity and registration accuracy [27].

### 3.4. Matrix Deposition Strategies

Matrix deposition must produce adequate surface coverage and matrix–analyte interaction without compromising the native lateral distribution of lipids. Common approaches include manual spraying, automated pneumatic spraying, electrospray coating, sublimation, thermal evaporation, dry-powder deposition, and post-deposition recrystallization. These methods differ in solvent exposure, extraction efficiency, crystal dimensions, coating thickness, and reproducibility [19,20,21].

Spray-based deposition is widely used because matrix concentration, solvent composition, flow rate, nozzle velocity, line spacing, substrate temperature, number of passes, and drying intervals can be adjusted systematically. Spray parameters must therefore balance lipid extraction against analyte migration and loss of plaque-scale spatial detail [19,20].

A direct comparison of automated spraying and sublimation for common positive-ion matrices showed that the optimal approach depends on the matrix, target lipid, tissue type, crystal morphology, and desired spatial resolution. Spraying can provide stronger extraction and higher signal for selected phospholipids, whereas sublimation generally produces finer crystals and lower solvent-driven delocalization [34].

A separate comparison within a fluorescence-compatible MALDI imaging pipeline similarly demonstrated that spray coating and sublimation yield different lipid-detection efficiencies. Performance varied with matrix chemistry, tissue composition, crystal size, ionization mode, and the use of conventional MALDI or MALDI-2 post-ionization, confirming that deposition methods cannot be evaluated independently of the complete analytical configuration [35].

Manual spraying remains accessible and inexpensive but is difficult to reproduce precisely. Automated deposition is preferable for comparative studies because operating parameters can be recorded, although humidity, nozzle condition, matrix age, and tissue temperature may still introduce batch effects [20,21,34].

Cluster-airflow-assisted matrix coating has been proposed as a strategy for producing fine and homogeneous matrix crystals for high-resolution imaging of lipids and small molecules. This approach can improve coating homogeneity and reduce local over-wetting in high-resolution experiments [36].

Sublimation deposits matrix vapour under reduced pressure and initially avoids contact between the tissue and liquid solvent. This reduces lateral diffusion and makes sublimation attractive for high-resolution imaging of small pathological structures. Its disadvantages include limited extraction of some lipid classes, restricted compatibility with volatile matrices, and possible variation in coating thickness across the deposition chamber [19,34,35].

Post-sublimation recrystallization can enhance matrix–analyte incorporation by exposing the dry layer to controlled solvent vapour. Properly optimized recrystallization improves ion yield while retaining much of the spatial fidelity achieved by sublimation. Excessive humidity, temperature, or exposure time can, however, enlarge crystals and mobilize tissue lipids [19,34].

Low-temperature thermal evaporation has been introduced as a controlled dry-deposition strategy. Deposition of 1,5-diaminonaphthalene by this method enabled imaging of polar lipids in sagittal mouse brain sections and provided control over matrix-layer formation while limiting solvent exposure [37].

Dry-deposition strategies, including sublimation, thermal evaporation, dry spraying, and powder-based coating, are increasingly used when cellular or near-cellular spatial resolution is required. Their principal advantage is reduced solvent-induced diffusion, whereas their principal limitation is often insufficient extraction of weakly accessible analytes. Controlled recrystallization, MALDI-2, or modified laser conditions may therefore be needed to recover sensitivity [38].

Matrix application should not be evaluated only by the number of detected peaks. Comparison of six matrices for 10 μm lipid imaging showed that matrix chemistry affects lipid-class coverage, spectral background, fragmentation, image contrast, and compatibility with on-tissue tandem mass spectrometry. A high peak count may include matrix clusters, redundant adducts, or fragments and does not necessarily indicate superior biological information [39].

Evaluation of a deposition method should include crystal size, layer uniformity, signal reproducibility, lipid-class coverage, tissue morphology, and analyte delocalization [20,39].

### 3.5. Spatial Fidelity, Reproducibility, and Quality Control

Spatial fidelity should be demonstrated experimentally rather than inferred from nominal pixel size or matrix crystal dimensions. Molecular delocalization may be indicated by signals extending beyond tissue boundaries, edge accumulation, loss of grey–white matter interfaces, disappearance of small anatomical structures, or increased similarity between normally distinct regions [17,20].

Technical reproducibility requires replicate sections, balanced slide layouts, randomized preparation order, and repeated analytical batches. Serial brain sections are not identical, particularly when amyloid plaques or other microscopic lesions are investigated. Processing all control samples in one batch and all AD specimens in another would therefore confound analytical and biological variation [20,21].

A lipid MALDI-MSI workflow linked to LC–MS-based quality control has been developed to improve monitoring of analytical variation and quantitative performance. Such approaches enable evaluation of extraction recovery, lipid-class coverage, calibration behaviour, and inter-batch stability rather than relying exclusively on visual assessment of ion images [40].

Pooled reference-tissue sections should be included across matrix-application and acquisition batches. Their spectra, representative lipid signals, adduct ratios, and anatomical contrast can reveal deposition failure or instrumental drift before AD–control comparisons are performed [20,40].

Internal standards can correct part of the variation caused by matrix heterogeneity, local ion suppression, and detector response. A multiclass internal-standard mixture applied to brain tissue improved normalization across glycerophospholipid and sphingolipid subclasses and supported comparisons among anatomically distinct brain regions [41].

Class-matched isotopically labelled standards are preferable because lipid subclasses differ in extraction, ionization, fragmentation, and adduct formation. However, standards applied onto the tissue surface do not fully reproduce the intracellular location, membrane binding, or extraction behaviour of endogenous molecules. Internal-standard normalization should therefore be considered an improvement in comparability rather than proof of absolute quantification [20,41].

Normalization based exclusively on total-ion current may introduce bias when several highly abundant lipids dominate the spectrum or when disease produces widespread changes in tissue composition. Reference-ion, median, root-mean-square, or internal-standard normalization may be more suitable in specific experiments, but the selected procedure should be validated and reported transparently [20,21,41].

Integration of MALDI-MSI with LC–MS/MS can combine spatial localization with broader molecular identification and compositional lipid analysis. A combined MALDI-MSI and LC–MS/MS workflow applied to whole *Caenorhabditis elegans* demonstrated that spatially resolved lipid signals can be linked with orthogonal lipid identifications obtained from extract-based analysis [42].

Orthogonal validation also helps determine whether several *m*/*z* features represent distinct lipids or alternative adducts, isotopes, and fragments of the same molecule. LC separation, high-resolution MS/MS, ion mobility, authentic standards, and histological co-registration provide complementary levels of molecular and spatial confidence [17,21,42].

### 3.6. Special Considerations for Human Brain and Alzheimer’s Disease Tissue

Human AD tissue presents additional preanalytical challenges because cortical atrophy, variable myelin content, vascular pathology, neuronal loss, plaque burden, and gliosis alter both tissue composition and ion suppression. Samples should be compared at equivalent anatomical levels, and grey matter, white matter, vasculature, and damaged tissue should be segmented where possible before statistical analysis [11,17].

White matter is enriched in cholesterol, sulfatides, sphingomyelins, and other myelin-related lipids, whereas cortical layers contain distinct glycerophospholipid and neuronal-membrane profiles. Differences in the proportions of these compartments may be mistaken for disease-associated lipid changes when anatomical sampling is inconsistent [17].

Fresh-frozen human brain remains preferable for broad untargeted lipid imaging, but archival formalin-fixed paraffin-embedded material may be the only available resource for rare clinical cohorts. Formalin fixation and paraffin processing extract or chemically modify a substantial fraction of the native lipidome, meaning that FFPE sections provide a selected residual lipid profile rather than a complete representation of the original tissue composition [43].

The use of 6-aza-2-thiothymine has demonstrated that selected lipid species can nevertheless be imaged in FFPE clinical sections in both ionization modes. These results broaden the potential use of archived samples but do not establish equivalence with fresh-frozen tissue. Fixation time, paraffin processing, storage duration, deparaffinization, washing, matrix chemistry, and polarity must be reported and interpreted as part of the analytical method [43].

Plaque-scale imaging requires sample preparation that preserves microscopic boundaries and supports accurate pathological registration. Combined lipid MSI and MALDI immunohistochemistry enabled spatial mapping of gangliosides, Aβ-related signals, and protein markers at cellular-scale resolution in an AD mouse model, illustrating the need for small matrix crystals, flat sections, controlled solvent exposure, and accurate image alignment [44].

Human amyloid plaques are molecularly heterogeneous and may vary among sporadic AD, Down syndrome-associated pathology, and different brain regions. High-quality fresh-frozen sections and precise histological co-registration were essential for reflector-mode MALDI-MSI analysis of Aβ proteoforms in human post-mortem brain, demonstrating the level of tissue preservation required for lesion-specific molecular interpretation [45].

Same-section multimodal analysis can reduce uncertainty caused by differences between adjacent sections. Sequential imaging of lipids, protein markers, and histology within the Aβ plaque microenvironment has been demonstrated using a benchtop MALDI platform, allowing direct comparison of molecular and pathological signals within the same tissue geometry [46].

The benefit of same-section analysis must be balanced against cumulative chemical interference. Matrix removal, staining solutions, antibody buffers, detergents, enzymatic treatments, and antigen-retrieval procedures can extract lipids or alter ionization. The most labile molecular layer should therefore be analysed first, which in spatial lipidomics generally means acquiring lipid MSI before aqueous histology or immunohistochemistry [27,44,46].

Adjacent-section designs avoid direct chemical interference but introduce registration uncertainty and may fail to capture the same plaque or vascular lesion in both sections. Same-section and adjacent-section workflows should therefore be selected according to lesion size, required molecular modalities, tissue availability, and tolerance for chemical processing [44,45,46].

The principal preanalytical and matrix-deposition factors affecting lipid preservation, spatial fidelity, and reproducibility are summarized in Table 2.

Collectively, these variables demonstrate that lipid MALDI-MSI data quality is determined by the cumulative performance of the entire preanalytical workflow rather than by any single preparation step. In AD studies, these controls are required to distinguish genuine region- and plaque-associated lipid remodeling from variation introduced by tissue handling, anatomical sampling, matrix deposition, or analytical batch effects. Among these variables, matrix chemistry and deposition exert particularly direct effects on lipid extraction, ionization, spectral background, and molecular coverage, and are therefore considered in detail in the following section.

## 4. Matrix Selection for Lipid MALDI-MSI

Matrix selection is a primary determinant of the analytical window obtained in lipid MALDI-MSI. The matrix absorbs laser radiation, participates in desorption and ion formation, and influences analyte extraction, proton transfer, deprotonation, cationization, fragmentation, crystal morphology, spectral background, and spatial resolution. Because lipid classes differ markedly in polarity, charge state, proton affinity, acidity, stability, and adduct-forming behavior, no single matrix provides uniform access to the complete tissue lipidome [19,20,21].

The consequences of matrix selection extend beyond analytical sensitivity. Preferential detection of phosphatidylcholines, sphingomyelins, acidic glycerophospholipids, glycosphingolipids, neutral lipids, sterols, or oxidized species determines which components of Alzheimer’s disease (AD)-associated lipid remodeling become visible. Accordingly, matrix-dependent selectivity must be regarded as a source of biological emphasis and analytical bias rather than merely a technical difference in signal intensity [17,20].

### 4.1. Physicochemical and Analytical Criteria for Matrix Selection

An effective MALDI matrix should absorb efficiently at the applied laser wavelength, support analyte desorption and ion formation, and generate limited chemical interference in the mass range of interest. For imaging applications, it must additionally form a homogeneous layer, produce crystals compatible with the selected pixel size, remain sufficiently stable under instrumental pressure, and preserve the lateral distribution of tissue molecules [19,21].

The relationship between matrix structure and analytical performance is multifactorial. Aromaticity and conjugation influence ultraviolet absorption, whereas acidic or basic functional groups affect proton-donor and proton-acceptor behavior. Volatility determines compatibility with sublimation, while solubility controls the choice of deposition solvents and the extent of analyte extraction. Crystal morphology, matrix-layer thickness, thermal stability, and tendency to form clusters further influence image quality and molecular coverage [19,47].

Leopold et al. reviewed developments in lipid-oriented MALDI matrices and emphasized that established compounds such as 2,5-dihydroxybenzoic acid (DHB), 9-aminoacridine (9-AA), 1,5-diaminonaphthalene (DAN), and norharmane retain distinct advantages and limitations rather than forming a simple hierarchy of performance [47]. Matrix suitability must therefore be defined in relation to the target lipid class, polarity, tissue composition, instrumental platform, and spatial resolution.

A systematic brain-tissue study by Lu et al. compared several matrices, deposition approaches, and acquisition polarities and showed that matrix performance changed with tissue type and analytical objective [48]. Recrystallized α-cyano-4-hydroxycinnamic acid performed favorably for selected positive-ion measurements, whereas 9-AA provided a different molecular window in negative mode. The study demonstrates that optimal matrix selection should be based on comparative experimental evidence rather than conventional laboratory preference [48].

Comparison of norharmane, 9-AA, DHB, and DAN has likewise shown that matrices generate substantially different lipid profiles from related tissue sections. Differences were observed in feature number, ion intensity, spatial pattern, and correspondence between alternative imaging approaches, confirming that the selected matrix can influence the apparent molecular architecture of the specimen [49].

Matrix evaluation should include spatial information as well as spectral yield. Shafer and Neumann compared common lipid-MSI preparation variables in a neuroimaging workflow compatible with immunofluorescence and demonstrated that matrix choice influences lipid coverage, image quality, tissue morphology, and downstream multimodal compatibility [50]. A matrix producing the highest number of peaks may therefore be unsuitable if it compromises microscopic localization or subsequent histopathological analysis.

The optimal matrix can also depend on the physiological state of the tissue. Validation of MALDI imaging in ex vivo brain slices showed that lipid profiles and image quality were influenced by the matrix–polarity combination, with DAN-based negative-ion analysis providing useful access to acidic brain lipids [51]. Such results caution against transferring a protocol between fresh-frozen brain, cultured slices, organoids, and post-mortem human tissue without validation.

### 4.2. Matrices for Positive-Ion Lipid Imaging

DHB remains one of the most commonly used matrices for positive-ion lipid imaging. It efficiently supports the detection of abundant phosphatidylcholines and sphingomyelins, which are observed predominantly as protonated, sodiated, or potassiated ions. Depending on additives and tissue cation content, DHB may also facilitate the detection of triacylglycerols, cholesteryl esters, and other neutral lipids as metal or ammonium adducts [10,19,47].

The widespread use of DHB reflects its commercial availability, established deposition procedures, compatibility with spraying and sublimation, and broad response for phosphocholine-containing species. However, DHB can produce heterogeneous crystals, low-mass matrix interference, multiple analyte adducts, and strong signals from abundant PC and SM species that suppress less efficiently ionized lipids [19,20,39].

DHB performance is highly dependent on deposition and recrystallization. Fine sublimated coatings favor high spatial resolution but may provide limited extraction, whereas wet spraying can increase lipid incorporation at the cost of greater lateral migration. Controlled recrystallization can improve sensitivity, but excessive solvent exposure enlarges crystals and may blur plaque-scale molecular distributions [34,35].

CHCA is more commonly associated with peptide and small-molecule analysis but can provide useful positive-ion lipid profiles under optimized conditions. Its relatively efficient energy transfer and tendency to form small crystals may support high-resolution imaging, although lipid-class coverage and fragmentation can differ from DHB-based analysis. In brain-tumor tissue, recrystallized CHCA produced analytically valuable metabolite and lipid information, emphasizing the importance of evaluating nontraditional matrix–application combinations [48].

Norharmane can also provide abundant positive-ion phospholipid and sphingolipid signals. In quantitative brain-MSI workflows, DHB and norharmane showed complementary class-dependent performance, demonstrating that matrix selection can alter both the detectable lipid repertoire and the effectiveness of internal-standard normalization [41].

Positive-ion spectra are strongly affected by sodium and potassium availability. The same phospholipid may generate [M + H]^+^, [M + Na]^+^, and [M + K]^+^ ions, whose relative intensities vary between anatomical regions. Matrix acidity, solvent composition, salts, and tissue pretreatment can redistribute signal among these forms, and interpretation based on a single adduct may consequently underestimate the underlying lipid distribution [10,20].

Neutral lipids remain difficult to image because they lack strongly acidic or basic functional groups. Salt-enhanced MALDI trapped-ion-mobility imaging has been developed to improve the sensitivity and specificity of neutral-lipid detection through controlled cationization [52]. This approach illustrates that matrix selection may need to be integrated with an intentionally introduced salt environment rather than optimized as an isolated chemical variable.

Salt-assisted strategies require careful control because increased cation abundance may improve one lipid group while suppressing another or generating multiple adduct families [52].

### 4.3. Matrices for Negative-Ion Lipid Imaging

Negative-ion MALDI-MSI is essential for acidic glycerophospholipids and glycosphingolipids. These include phosphatidylinositols, phosphatidylserines, phosphatidylglycerols, phosphatidic acids, sulfatides, gangliosides, and selected ceramides and fatty acids. Because many of these classes are directly relevant to neuronal membranes, myelin, lysosomal pathways, and amyloid-plaque biology, negative-ion matrix selection is particularly important in AD research [17,19].

9-AA is a widely used basic matrix that supports deprotonation of acidic lipids and frequently provides relatively low background in relevant parts of the lipid mass range. It has been used to visualize PI, PS, PG, PA, sulfatides, and other anionic species. Nevertheless, its vacuum stability, crystallization, deposition reproducibility, and performance at very high spatial resolution can be limiting [19,47].

DAN is another established negative-ion matrix for brain lipidomics. It supports the detection of PE, PI, PS, sulfatides, ceramides, fatty acids, and related species and is compatible with sublimation-based fine coatings. Its disadvantages include oxidation, matrix-derived background, variable vacuum stability, and matrix- and fluence-dependent fragmentation [17,26].

The optimal negative-ion matrix may vary even within one phospholipid family. Dabija et al. compared matrices for phosphatidylinositol and phosphoinositide analysis and found major differences in sensitivity, image quality, and molecular coverage [53]. This is analytically important because low-abundance phosphorylated PI species may not follow the same matrix-response pattern as abundant PI lipids.

Specialized matrix chemistry can improve classes that are poorly represented by conventional compounds. A DBDA-based matrix increased signals from free fatty acids and sulfatides while limiting interference in parts of the negative-ion mass range [54]. Such class-selective matrices are valuable when the biological question is narrowly defined, but they should not be interpreted as providing comprehensive lipidomic coverage.

Acetophenone-derived matrices have also been used for neuropathogenic sphingolipid imaging. DHA-based workflows have supported spatial mapping of glycosphingolipids in nervous tissue, although volatility, sublimation behavior, coating stability, and fragmentation must be controlled [55].

The balance between sensitivity and structural preservation is especially important for acidic glycosphingolipids. Gangliosides may undergo loss of sialic-acid residues, while sulfatides can produce sulfate-related fragments. A matrix that yields high total signal but extensive decomposition may create artifactual distributions of simpler glycosphingolipid ions [17,26]. This risk is directly relevant to the interpretation of GM2-, GM3-, and sulfatide-related signals in plaque-associated regions.

### 4.4. Dual-Polarity Matrices

Dual-polarity matrices enable complementary positive- and negative-ion acquisitions from the same tissue section. This approach reduces the anatomical mismatch inherent to adjacent-section analysis and can link phosphocholine-rich profiles with acidic phospholipid and glycosphingolipid distributions within the same tissue geometry [22,23].

Norharmane is among the most established broad-polarity matrices for brain lipid imaging. It can support PC and SM detection in positive-ion mode and PI, PS, sulfatides, gangliosides, and related classes in negative-ion mode. It is also compatible with sublimation and sequential multiomic workflows, making it attractive when tissue availability is limited [17,27].

Broad polarity does not imply uniform response. Norharmane may differ markedly from DHB, DAN, or 9-AA in signal intensity, lipid-class representation, background, precursor survival, and adduct formation. Its performance must therefore be assessed independently for each polarity and target class [26,39,47].

NEDC has emerged as a high-performance dual-polarity matrix. Chen et al. demonstrated 5 μm dual-polarity imaging without matrix reapplication, achieving complementary lipid detection at single-cell or subcellular spatial scales [22]. The study illustrates how matrix chemistry, deposition, and acquisition optimization must be coordinated to maintain both signal intensity and spatial resolution.

Aminated cinnamic acid analogues provide another rationally designed dual-polarity matrix family. These compounds combine ultraviolet absorption with proton-donating and proton-accepting functionality and have enabled high-resolution imaging of polar lipids and metabolites [23].

Nitroindole derivatives have also been introduced as dual-polarity matrices for complex biological samples. Structural modification within this family altered ionization efficiency, background, crystal behavior, and the relative performance of positive- and negative-ion detection [56].

Basic Blue 7 represents a multifunctional dual-polarity matrix that simultaneously stains tissue and supports lipid imaging. In mouse brain, it produced clean negative-ion spectra and useful signals from several lipid classes while enabling optical inspection of the stained section before acquisition [57]. This approach may facilitate histological annotation and MSI co-registration, although immersion-based application must be evaluated for possible analyte extraction or migration.

Single-cell lipidomics integrating dual-polarity ionization with ion mobility has further shown that polarity switching and gas-phase separation provide complementary information [58]. Positive and negative modes emphasize different lipid classes, while ion mobility helps resolve isobaric or isomeric ions that remain overlapped in conventional MS1 data.

Dual-polarity strategies can also be implemented serially rather than through one universal matrix. Serial matrix replacement and repeated imaging may expand molecular coverage and permit targeted MS/MS measurements, but each matrix-removal and reapplication step introduces risks of analyte loss, registration error, tissue deformation, and cumulative contamination [59].

Multimodal same-section MSI provides another use case for broad-polarity matrices. Zhan et al. described multimodal analysis in which norharmane enabled positive- and negative-ion molecular imaging from one tissue section before further analytical steps [60]. Such designs are particularly relevant to AD, where direct registration of complementary lipid signals with pathology is desirable.

### 4.5. Emerging and Reactive Matrices for Underrepresented Lipid Classes

Conventional matrices are optimized primarily for abundant polar lipids and often perform poorly for sterols, neutral storage lipids, low-abundance oxidized phospholipids, and structurally similar lipid isomers. Emerging matrices increasingly address these limitations through selective chemical reactivity, controlled cationization, or improved absorption and crystallization [47].

Oxidized lipids are analytically challenging because they occur at low abundance, encompass heterogeneous functional groups, and may be unstable during sample handling and laser irradiation. Prabutzki et al. evaluated conventional matrices together with 1-pyrenebutyric hydrazide for the detection of oxidized major phospholipids [61]. Hydrazide derivatization improved access to selected carbonyl-containing oxidation products and provides a route toward future spatial imaging of oxidative lipid pathology.

This capability is relevant to AD because lipid peroxidation accompanies oxidative stress, mitochondrial dysfunction, neuroinflammation, and membrane injury. However, reactive-matrix workflows must be validated for reaction yield, specificity, tissue penetration, side products, and possible analyte migration before differences in image intensity can be interpreted biologically [61].

Norharmane can also act as a photoreactive reagent rather than only as a conventional proton-transfer matrix. Light-reactive derivatization has been used to obtain information on double-bond location in unsaturated lipids, providing structural discrimination beyond sum-composition assignments [25]. Such approaches may help distinguish lipid isomers with different biological functions but identical nominal and exact masses.

Cholesterol is poorly ionized by many standard organic matrices because it has low polarity and lacks a readily ionizable group. A p-nitroaniline-based method enabled direct and rapid visualization of cholesterol in AD and cancer tissue [62]. This targeted matrix strategy illustrates that sterol imaging may require fundamentally different ionization chemistry from phospholipid imaging.

Neutral-lipid analysis similarly benefits from cation-enhancing additives and ion-mobility separation. Salt-enhanced MALDI-TIMS has improved the detection of neutral lipids while reducing ambiguity from overlapping ions [52]. Nevertheless, deliberate salt addition modifies the ionization environment and should be validated against untreated sections and orthogonal lipidomic measurements.

ATT has shown potential for lipid profiling in both polarities, including analysis of FFPE clinical sections [43]. Its use in chemically preserved tissue is relevant because conventional matrices may provide insufficient residual-lipid coverage after fixation and paraffin processing. However, matrix optimization cannot restore lipid species already extracted or chemically altered during tissue processing [43].

Novel matrix screening continues to identify compounds with broader or class-specific lipid performance. Screening of small aromatic molecules has demonstrated that nitroaniline and related structures vary markedly in ultraviolet absorption, ionization efficiency, background, and lipid-image quality [63].

A recent broader assessment of MALDI matrix innovation has emphasized the movement from empirically selected small aromatic acids toward functional matrices, nanomaterials, reactive matrices, binary systems, and compounds designed for specific ionization pathways [64]. For lipid MSI, the main value of these innovations lies in expanding access to underrepresented chemical space rather than simply increasing total peak counts.

### 4.6. Matrix-Dependent Lipid Coverage and Analytical Bias

Matrix choice creates class-dependent detection bias. DHB-based positive-ion analysis commonly emphasizes PC and SM, whereas 9-AA-, DAN-, and norharmane-based negative-ion experiments provide greater access to PI, PS, PE, sulfatides, and gangliosides. Results obtained with these approaches are complementary but not directly interchangeable [17,19,47].

The extent of bias depends on tissue composition. Human brain white-matter tracts contain regionally distinct mixtures of myelin-associated lipids, and spatial lipidomics has demonstrated differences not only between grey and white matter but also among individual white-matter structures [65]. A matrix favoring sulfatides and other acidic myelin lipids will represent these differences differently from a positive-ion matrix dominated by PC and SM signals.

Human brain organoids provide an experimentally accessible model for investigating spatial metabolic and lipid heterogeneity in a three-dimensional neural context. Mass spectrometry imaging protocols developed for human brain organoids demonstrate that molecular distributions can be mapped while retaining regional tissue architecture, supporting their use in neurodevelopmental and neurodegenerative disease research [66].

Disease-associated changes in tissue composition also affect matrix response. Lipid imaging in a model of breast-cancer brain metastasis demonstrated distinct lipid patterns related to ischemic or hypoxic tissue environments [67]. Although not an AD study, it shows that necrosis, edema, hypoxia, and altered membrane density can modify both the true lipidome and the matrix-dependent ionization environment.

Matrix-related ion suppression is particularly relevant in heterogeneous brain tissue. Abundant phosphocholine lipids can dominate positive-ion spectra, whereas abundant sulfatides and PI species can dominate negative-ion spectra. The resulting image intensity depends on local competition for desorption and charge, meaning that between-region comparisons may not be proportional to concentration [20,41].

Adduct formation adds another source of apparent molecular complexity. A single lipid may produce protonated, deprotonated, sodium-, potassium-, ammonium-, or matrix-associated ions. If these forms are annotated independently, one compound may be counted repeatedly, while anatomical differences in salt content may be mistaken for changes in lipid abundance [10,20].

Matrix-dependent fragmentation can produce an even more serious interpretive error. Cousineau et al. demonstrated that matrices differ in the degree of in-source phospholipid fragmentation and that fragmentation varies with experimental conditions [26]. Monitoring precursor-to-fragment relationships should therefore form part of matrix validation, especially when fragment ions overlap with plausible endogenous lipid species.

This issue is critical for sphingolipids and glycerophospholipids that share common fragments or neutral losses. Ion mobility, on-tissue MS/MS, LC–MS/MS, authentic standards, and analysis at multiple laser fluences can increase identification confidence but do not eliminate matrix-induced bias at the ionization stage [21,26].

Matrix performance should consequently be evaluated using several complementary criteria: representative lipid-class coverage, precursor survival, background intensity, adduct complexity, crystal dimensions, coating uniformity, spatial fidelity, technical reproducibility, compatibility with MS/MS, and agreement with orthogonal lipidomics. Total peak count alone is insufficient [39,48]. The principal characteristics of established, dual-polarity, and specialized matrices relevant to AD lipid imaging are compared in Table 3.

**Table 3:** Comparative characteristics of matrices used in lipid MALDI-MSI and their relevance to Alzheimer’s disease research.

Matrix	Ion Mode	Preferential Lipid Classes	Main Relevance and Limitation in AD
DHB	Positive	PC, SM; selected neutral-lipid adducts	Widely used for membrane phospholipids and multimodal plaque imaging; may produce heterogeneous crystals, multiple adducts, and suppression of less efficiently ionized lipids [10,19,20,34,35,39,47].
CHCA	Predominantly positive	Selected phospholipids, metabolites, and small molecules	Supports small crystals and high-resolution imaging, but lipid coverage and fragmentation depend strongly on recrystallization; AD-specific validation remains limited [48].
9-AA	Negative	PI, PS, PG, PA, sulfatides	Useful for acidic phospholipids and myelin-related sulfatides; vacuum stability, crystallization, and very-high-resolution performance may be limiting [19,47,48,53].
DAN	Predominantly negative	PE, PI, PS, sulfatides, ceramides, fatty acids	Relevant to acidic brain lipids and ceramide-related species; oxidation, background, and fluence-dependent fragmentation require control [17,26,51].
Norharmane	Dual polarity	PC and SM in positive mode; PI, PS, sulfatides, and gangliosides in negative mode	Enables same-section comparison of complementary lipid classes and multimodal workflows; response and fragmentation differ between polarities [17,25,26,27,39,41,47,49,60].
NEDC/aminated cinnamic acid analogues	Dual polarity	Polar phospholipids and metabolites	Support high-resolution dual-polarity imaging without matrix reapplication; performance remains tissue-, deposition-, and target-dependent [22,23].
DBDA/DHA-based matrices	Negative	Free fatty acids, sulfatides, glycosphingolipids	Useful for sulfatide depletion, myelin pathology, and ganglioside-related mechanisms; class coverage is narrow and coating stability or fragmentation may be limiting [54,55].
p-Nitroaniline-based matrices	Target-dependent	Cholesterol and selected sterols	Provide access to cholesterol poorly detected by conventional matrices; narrow target range limits broad untargeted lipidomic interpretation [62,63].
Reactive hydrazide matrices	Target-dependent	Carbonyl-containing oxidized phospholipids	Relevant to oxidative stress and membrane injury in AD; reaction specificity, yield, side products, and analyte migration require validation [61].
ATT	Dual polarity	Selected residual lipids in fresh-frozen and FFPE tissue	May extend analysis to archival AD cohorts, but cannot recover lipids removed or modified during fixation and paraffin processing [43].

### 4.7. Matrix Selection in Alzheimer’s Disease Studies

AD-associated lipid pathology encompasses phospholipids, sphingolipids, gangliosides, sulfatides, cholesterol, neutral lipids, fatty acids, and oxidized species. Because these groups require different ionization pathways, no single matrix–polarity combination can provide a complete representation of AD lipid remodeling [11,17].

A dual-polarity 4-aminocinnoline-3-carboxamide matrix was applied to a transgenic AD mouse model and provided broad access to metabolic alterations in both ion modes [68]. Compared with conventional polarity-specific matrices, this approach increased the range of detected molecular features and illustrated the value of broad-polarity analysis for complex neurodegenerative pathology.

Negative-ion MALDI-MSI of APP/PS1 brain disclosed decreases in sulfoglycosphingolipid and glycerophosphoinositol species in cognition-related regions [69]. These findings depended on conditions suitable for anionic lipid detection and demonstrate why a positive-ion phosphocholine-dominated experiment would provide an incomplete representation of AD-related changes.

Matrix selection is equally important for plaque-associated gangliosides. Co-registration of MALDI-MSI and histology has shown that GM2- and GM3-related signals localize to amyloid plaques [15]. Subsequent work connected plaque accumulation of these gangliosides with altered ganglioside degradation pathways, strengthening the interpretation that the images reflect localized metabolic remodeling rather than passive lipid adsorption [70].

Ganglioside measurements require particular attention to fragmentation. Loss of sialic acids can transform complex gangliosides into ions resembling simpler species, potentially inflating apparent GM2 or GM3 abundance. Negative-ion matrix conditions, low-fluence acquisition, ion mobility, and orthogonal analysis are therefore necessary for reliable plaque-level interpretation [17,26].

Trapped-ion-mobility MSI has revealed that structurally distinct amyloid plaques are associated with different lipid accumulations [71]. Ion mobility improves separation of overlapping lipid species, but the detected molecular population remains determined by the selected matrix, polarity, and desorption–ionization conditions.

Single-plaque analysis combining amyloid and lipid information used a DHB-based workflow to relate Aβ composition to ganglioside distributions [16]. This illustrates that matrix selection may also be constrained by compatibility with serial or multimodal measurements rather than by maximum lipid coverage alone.

Cellular-resolution mapping of plaque-associated gangliosides and protein markers has further demonstrated the need for fine matrix crystals and minimal lateral diffusion [44]. At 5 μm-scale imaging, matrix homogeneity and crystal dimensions become inseparable from the biological question because plaque-associated microdomains may be similar in size to the analytical sampling area.

Three-dimensional molecular imaging integrated with immunohistochemistry has been used to compare lipid and protein distributions across serial sections from AD-model brain [72]. Reproducible matrix deposition is essential in such experiments because section-to-section variation in coating density, extraction, or crystal size can create artificial three-dimensional gradients.

Multimodal lipid–protein–histology analysis has also identified plaque-associated gangliosides and phosphatidylinositols in APPPS1 brain [46]. The matrix in this setting must provide suitable lipid ionization while remaining removable or compatible with subsequent histology and immunodetection.

Human white-matter heterogeneity adds another interpretive layer. Regional differences among white-matter tracts can affect sulfatide, sphingomyelin, and phospholipid profiles independently of AD [65]. Matrix selection and anatomical sampling must therefore be considered jointly when comparing human AD and control brain.

Sulfatide imaging is particularly relevant because sulfatide depletion has repeatedly been associated with AD and myelin dysfunction. Recent MALDI-MSI applications in neuroinflammatory and demyelinating models have refined negative-ion approaches for localizing sulfatide species in central nervous system tissue [73]. These methods provide analytical guidance for future AD studies but require disease-specific validation.

Orthogonal quantitative measurements remain necessary for acidic glycosphingolipids. UHPLC–MRM-MS analysis of gangliosides and sulfatides in human brain has demonstrated disease-associated changes and provides a means of validating class-level trends observed by MALDI-MSI [74]. Imaging and extract-based measurements answer different questions, but their integration helps distinguish matrix-dependent signal variation from true abundance differences.

### 4.8. Practical Matrix-Selection Framework

Matrix selection should begin with the biological question. DHB is a rational starting point for abundant PC, SM, and selected neutral-lipid adducts in positive mode. Negative-ion matrices such as 9-AA, DAN, norharmane, DHA, or specialized basic compounds are more appropriate for PI, PS, PA, sulfatides, gangliosides, and related acidic species [17,19,47].

When broad lipid coverage is required, complementary positive- and negative-ion experiments should be planned from the outset. Dual-polarity matrices such as norharmane, NEDC, aminated cinnamic acid analogues, nitroindole derivatives, and Basic Blue 7 can reduce anatomical mismatch, but each polarity must still be evaluated independently for response, fragmentation, and class coverage [22,23,56,57].

The required spatial scale should determine acceptable crystal morphology and solvent exposure. Regional brain mapping may tolerate stronger wet extraction, whereas plaque-level and cellular imaging require fine, uniform coatings and strict control of analyte migration. Sublimation, dry deposition, controlled recrystallization, or optimized fine-droplet spraying may be preferable for these applications [34,35,38].

Underrepresented targets require dedicated strategies. Cholesterol may require a specialized matrix or derivatization, oxidized phospholipids may benefit from reactive hydrazides, neutral lipids may require controlled cationization, and double-bond localization may require photochemical or gas-phase structural methods [25,52,61,62].

Candidate matrices should be compared using standards and representative tissue sections. Evaluation should include class-specific sensitivity, spectral background, precursor survival, adduct distribution, crystal size, image contrast, spatial fidelity, repeatability, and compatibility with tandem MS and downstream pathology [26,39,48].

For broad AD spatial lipidomics, a minimum design should consider at least one positive-ion and one negative-ion acquisition. This is necessary to represent phosphocholine lipids, acidic glycerophospholipids, gangliosides, sulfatides, cholesterol-related species, and oxidized lipids, which occupy different chemical and pathological compartments [15,16,17,61,62,69,70,71].

The matrix-dependent relationship between lipid-class coverage, analytical performance, and AD-specific biological interpretation is summarized in Table 3.

Matrix selection should be reported as a justified analytical decision. Studies should specify matrix identity and purity, concentration, solvent composition, additives, deposition method, coating density, recrystallization conditions, polarity, laser settings, target lipid classes, and validation procedures. This information is necessary for reproducing experiments and distinguishing true AD-associated lipid remodeling from matrix-dependent analytical bias [20,21,47,48].

## 5. Spatial Lipid Pathology in Alzheimer’s Disease

Alzheimer’s disease is accompanied by extensive disruption of brain lipid homeostasis, but these alterations are not uniformly distributed throughout the tissue. Lipid remodeling occurs at several spatial scales, ranging from whole brain regions and cortical layers to white-matter tracts, individual amyloid-β (Aβ) plaques, dystrophic neurites, and plaque-associated glial microenvironments. MALDI-MSI and complementary spatial mass spectrometry methods have demonstrated that regional lipid abundance, plaque-associated enrichment, and local lipid depletion can coexist within the same brain section [11,17,75].

Spatial interpretation is essential because homogenate lipidomics averages signals from neurons, astrocytes, microglia, oligodendrocytes, myelin, vasculature, and extracellular pathological deposits. A lipid that appears only moderately altered in bulk tissue may be strongly enriched within a plaque or depleted within a narrow peri-plaque zone. Conversely, a regional change detected in a tissue extract may reflect altered proportions of grey matter, white matter, or gliosis rather than a uniform change in individual cells [17,75]. The biological significance of these patterns depends not only on their anatomical location but also on their position within disease progression, because early compensatory lipid responses may differ from changes associated with mature plaques, tau-related neurodegeneration, and advanced neuronal loss.

### 5.1. Regional and Spatiotemporal Lipid Remodeling in the AD Brain

One of the principal findings of spatial lipidomics is that AD-related lipid remodeling is region-dependent. The hippocampus, cerebral cortex, thalamus, corpus callosum, entorhinal cortex, basal forebrain, and white-matter tracts differ in cellular composition and baseline lipid profiles and therefore do not respond identically to amyloid pathology [17,69].

Li et al. applied spatial–temporal lipidomics to follow lipid dysregulation during AD progression in mouse brain and demonstrated that the magnitude and direction of lipid alterations changed with both disease stage and anatomical region [76]. The results indicate that the AD lipidome is dynamic rather than a fixed end-stage signature and that early compensatory responses may differ from changes observed after extensive plaque deposition.

Spatiotemporal analysis is particularly important because individual lipid classes may show non-linear trajectories. Membrane phospholipids, sphingolipids, fatty acids, and neutral storage lipids can increase at one stage and decline later as neuronal loss, glial activation, myelin injury, and lysosomal dysfunction progress. Cross-sectional comparisons at a single age may therefore capture only one phase of a broader pathological process [76].

Available evidence supports a provisional temporal model in which early-stage lipid remodeling may reflect altered membrane turnover, compensatory redistribution of phospholipids and cholesterol, and initial glial responses before extensive plaque accumulation. With increasing amyloid burden, ganglioside processing, lysosomal lipid accumulation, ceramide-related remodeling, and peri-plaque phospholipid changes become more prominent. At later stages, progressive neuronal loss, myelin disruption, chronic gliosis, and tau-associated degeneration may contribute to broader regional depletion or redistribution of membrane and myelin-related lipids. This sequence remains an interpretative framework rather than a fully established linear pathway because most available studies are cross-sectional and use different models, brain regions, and analytical workflows [11,17,76].

Spatial multiomic analysis of post-mortem human AD brain has similarly revealed regional differences in lipid and protein alterations. Toyama et al. integrated MALDI-MSI with LC–MS/MS-based lipidomic and proteomic analysis and demonstrated that molecular changes varied between anatomically and pathologically distinct tissue areas [77]. These findings support region-resolved sampling rather than treating an entire cortical block as a molecularly uniform specimen.

Three-dimensional analysis across serial brain sections further shows that lipid abnormalities may vary with both anatomical depth and local pathological burden. The integrated MALDI-MSI and immunohistochemistry workflow developed by Lu et al. revealed depth- and region-dependent changes in phosphatidylcholines, phosphatidylethanolamines, and other lipid classes in AD-model brain [72]. Such results indicate that a single two-dimensional section may not fully represent the molecular extent of a lesion.

A broader spatial map of the brain metabolome, lipidome, and glycome has demonstrated that molecular distributions follow anatomical boundaries and differ between wild-type and neurodegenerative-disease models [78]. Integration of several molecular layers helps distinguish lipid changes associated with general regional identity from those specifically related to amyloid or tau pathology.

The importance of regional baseline composition is also supported by high-resolution mapping of the healthy and diseased nervous system. Spatial lipidomics of human white-matter tracts demonstrated substantial differences among individual tracts, not merely between grey and white matter [65]. Consequently, comparisons between AD and control cases must account for tract identity and the proportion of myelinated tissue within each sampled region.

### 5.2. Amyloid Plaque-Associated Lipid Microenvironments

Amyloid plaques are surrounded by highly organized molecular microenvironments containing fibrillar Aβ, dystrophic neurites, activated microglia, reactive astrocytes, damaged membranes, lysosomal material, and locally redistributed lipids. Spatial neurolipidomics at the level of individual human plaques has shown that these structures are associated with both lipid enrichment and lipid depletion, rather than a uniform accumulation of membrane material [75].

Michno et al. performed single-plaque spatial neurolipidomics in post-mortem human AD brain and identified nearly 40 sphingolipid and phospholipid species with plaque-associated distributions [75]. Gangliosides, ceramide-related species, and selected glycerophospholipids were enriched in or around plaques, whereas several sulfatides displayed local depletion. These patterns demonstrate that individual lipid pathways occupy distinct plaque compartments.

Mechanistically, these spatial patterns may represent sequential and overlapping processes rather than independent observations. Membrane damage and dystrophic neurite formation can increase the local availability of phospholipids and sphingolipids; microglial phagocytosis and lysosomal processing may subsequently promote accumulation of simpler gangliosides and lysosomal lipids; astrocytic and inflammatory responses may contribute lysophospholipids and oxidized species; and progressive myelin or oligodendrocyte dysfunction may produce local or regional sulfatide depletion. MALDI-MSI identifies the anatomical convergence of these processes but does not by itself establish their causal order [44,75,79].

Multimodal MSI in AD mouse models has further resolved plaque-specific and region-specific lipid signatures. Trinklein et al. combined fluorescence microscopy, MALDI-2, ion mobility, and computational image analysis to associate individual lipid ions with plaques and defined anatomical regions [79]. This approach showed that plaque-associated lipid changes can differ among brain regions and between experimental models.

Nano-DESI MSI combined with immunofluorescence has independently demonstrated phospholipid accumulation within and around Aβ plaques [80]. Because nano-DESI does not require a MALDI matrix, agreement between MALDI-based and ambient-ionization findings strengthens the conclusion that plaque-associated phospholipid enrichment is biological rather than solely a matrix-dependent artifact.

Co-registration of MALDI-MSI with histology has shown that plaque-associated lipid signals can be aligned directly with Aβ deposits. Ollen-Bittle et al. demonstrated co-localization of GM2- and GM3-related ganglioside ions with amyloid plaques and validated an imaging pipeline for distinguishing plaque-associated lipids from broader regional differences [15].

At cellular spatial resolution, Good et al. simultaneously mapped gangliosides, Aβ peptides, and protein markers associated with microglia [44]. GM2 and GM3 accumulated within plaque microenvironments, indicating a relationship among amyloid deposition, altered ganglioside processing, and the recruitment or activation of glial cells.

Multimodal lipid–protein–histology imaging has also identified phosphatidylinositol species and gangliosides at plaque sites in APPPS1 mouse brain [46]. Same-section analysis reduces uncertainty arising from adjacent-section registration and supports direct correspondence between lipid signals, Aβ, and histopathological features.

Plaques are themselves heterogeneous. Trapped-ion-mobility MSI demonstrated that distinct amyloid plaque morphologies are associated with different lipid accumulations [71]. Plaque structure, maturation state, compactness, and surrounding cellular response may therefore determine the composition of the local lipid microenvironment. Accordingly, lipid distributions should be interpreted in relation to plaque maturation rather than treating all Aβ deposits as equivalent lesions.

### 5.3. Gangliosides, Sulfatides, Ceramides, and Sphingomyelins

Gangliosides are among the most consistently observed plaque-associated lipid classes. Simple gangliosides, particularly GM2 and GM3, are enriched around amyloid plaques in several mouse models and in human AD tissue [15,44,75]. Their accumulation may reflect lysosomal processing of more complex neuronal gangliosides, uptake of membrane debris by microglia, or altered glycosphingolipid catabolism.

Wang et al. linked plaque-associated GM2 and GM3 accumulation to elevation of the ganglioside degradation pathway in a transgenic mouse model [70]. This mechanistic connection suggests that plaque gangliosides are not merely components of damaged membranes but products of altered cellular lipid processing within the pathological niche.

Gangliosides may also interact directly with Aβ. GM1-rich membrane domains can influence Aβ conformation and aggregation, whereas the simpler gangliosides observed within mature plaques may reflect downstream degradation and lysosomal dysfunction. Spatial MSI cannot alone determine causality, but it can identify where these processes converge anatomically [11,79].

Sulfatides show a contrasting spatial behavior. MALDI-MSI of APP/PS1 mouse brain revealed decreased sulfoglycosphingolipid species in cognition-related regions, together with changes in glycerophosphoinositols [69]. Single-plaque human analysis also found depletion of selected sulfatides near amyloid deposits [75].

Sulfatide loss may indicate myelin disruption, oligodendrocyte dysfunction, altered apolipoprotein-mediated transport, or increased degradation. Because sulfatides are strongly enriched in myelin, regional or plaque-associated depletion must be interpreted together with white-matter content and local tissue architecture [65,69]. APOE-dependent lipid transport may contribute to this relationship because apolipoprotein-mediated redistribution links glial lipid handling, cholesterol transport, myelin-associated lipids, and the response to amyloid pathology.

Studies in other neurodegenerative and demyelinating models demonstrate that individual sulfatide species respond differently according to acyl-chain length and hydroxylation [73,81]. These observations are relevant to AD because class-level measurements may obscure opposing changes among molecular species with different cellular origins and membrane functions.

Ceramide and sphingomyelin metabolism is also dysregulated in AD. Multiomic analysis identified the ceramide/sphingomyelin pathway as a disease-associated network and potential therapeutic target, linking lipid species with genetic, transcriptomic, and clinical data [82]. Ceramide accumulation may promote apoptosis, inflammation, membrane reorganization, and amyloidogenic processing.

Single-plaque MSI has localized ceramide-1-phosphates and ceramide phosphoethanolamine species to plaque-associated regions [75]. Their distribution differs from that of sulfatides and gangliosides, emphasizing that sphingolipid subclasses undergo distinct spatial remodeling rather than a uniform pathway-wide increase.

Experimental membrane studies indicate that AD-like changes in ceramide and sphingomyelin composition can reorganize neuronal plasma-membrane microdomains and alter interactions with cholesterol [83]. Such biophysical effects provide a plausible mechanism through which spatially restricted sphingolipid changes could influence receptor signaling, membrane curvature, and amyloidogenic processing.

### 5.4. Glycerophospholipid Remodeling

Glycerophospholipids form the structural basis of neuronal and glial membranes and are extensively remodeled in AD. Spatial MSI studies have reported changes in phosphatidylcholines, phosphatidylethanolamines, phosphatidylinositols, phosphatidylserines, phosphatidic acids, lysophospholipids, and plasmalogens [17,72,75].

Plaque-associated phosphatidylinositols have been detected in multimodal MALDI-MSI studies of APPPS1 tissue [46]. PI species may originate from damaged neuronal membranes, activated glial cells, endolysosomal compartments, or signaling pathways enriched near plaques. Their negative-ion detection also illustrates why dual-polarity imaging is necessary for comprehensive plaque characterization.

Nano-DESI imaging has shown accumulation of several phospholipids both inside and immediately surrounding plaques [80]. The peri-plaque distribution suggests that lipid remodeling extends beyond the insoluble amyloid core into zones containing dystrophic neurites, reactive glia, and altered membrane turnover.

Glycerophospholipid remodeling in AD is influenced by both fatty-acid composition and anatomical region. Dietary DHA supplementation in wild-type and Tg2576 mice produced selective changes in phospholipid species across different brain regions, demonstrating that cerebral phospholipid responses are neither uniform nor adequately represented by whole-brain measurements [84]. These findings also indicate that disease genotype can modify the incorporation and redistribution of polyunsaturated fatty acids within membrane lipids.

Such regional specificity is biologically important because the fatty-acyl composition of phospholipids influences membrane fluidity, susceptibility to oxidation, phospholipase activity, and the generation of lipid mediators. Consequently, class-level or whole-tissue measurements may conceal molecular changes that occur selectively in vulnerable anatomical compartments [84].

Post-mortem lipidomics has identified distinct alterations in cerebral cortex and white matter during sporadic AD progression [85]. Although extract-based rather than imaging-based, these results provide orthogonal evidence that phospholipid and sphingolipid remodeling differs among tissue compartments and support anatomically stratified interpretation of MSI datasets.

Lysophospholipids may represent membrane hydrolysis, oxidative injury, or activated phospholipase pathways. Recent work has shown that lysophosphatidylcholine and lysophosphatidylethanolamine can accumulate around amyloid pathology in association with astrocyte activation and oxidative stress, partly independently of microglial activity [86]. Their presence therefore may identify a different cellular component of the plaque niche than gangliosides or lysosomal lipids.

Direct spatial evidence linking glycerophospholipid remodeling to tau pathology remains less developed than evidence for amyloid-associated microenvironments. Tau-mediated cytoskeletal disruption, axonal degeneration, synaptic loss, and neuronal membrane breakdown provide plausible sources of regionally altered phospholipid and lysophospholipid profiles, but these mechanisms require validation through co-registration of lipid MSI with tau-specific neuropathological markers.

### 5.5. Cholesterol, Cholesteryl Esters, and Neutral Lipids

Cholesterol is essential for synaptic membranes, myelin, lipid rafts, and amyloid precursor protein processing, but it is difficult to detect using conventional phospholipid-oriented MALDI matrices. Specialized matrix and metal-assisted strategies have therefore been developed to visualize its tissue distribution [62].

Direct MALDI-MSI using p-nitroaniline revealed regional cholesterol distributions in AD-model mouse brain and demonstrated differences among cortical, thalamic, and white-matter structures [62]. The study established that targeted ionization chemistry can provide spatial information on sterols that is largely absent from standard lipid-MSI datasets.

Reactive matrix chemistry offers an alternative strategy. Yang et al. developed reactive matrices for sensitive MALDI-MS analysis and imaging of cholesterol in brain tissue [87]. Derivatization improves ionization by introducing a readily detectable charged or chargeable group, although reaction efficiency and tissue penetration must be controlled.

Silver-assisted laser desorption/ionization MSI has also been used to investigate cholesterol in amyloid-containing tissues. Thermal evaporation of silver provides a uniform metal layer and enables formation of cholesterol–silver ions with limited wet-solvent exposure [88]. Such workflows are useful for testing whether cholesterol accumulates directly within amyloid plaques or follows broader membrane-rich structures.

Cholesteryl esters and triacylglycerols represent storage forms that may accumulate in lipid droplets of reactive glia. A neurodegenerative-disease lipidomics resource has shown that APOE4 can promote cholesteryl-ester accumulation in human astrocytes and that CE abundance is increased in AD brain datasets [89]. Spatial localization of these species will be important for connecting APOE genotype, astrocyte lipid storage, and plaque-associated inflammation.

APOE ε4 should therefore be considered not only as a genetic risk factor but also as a modifier of the spatial lipid environment. Altered astrocytic cholesterol handling, lipid-droplet formation, and redistribution of cholesteryl esters may influence neuronal lipid supply, glial responses, and the composition of plaque-associated tissue. However, direct genotype-stratified MALDI-MSI evidence in human AD remains limited.

Neutral-lipid imaging remains analytically less mature than phospholipid or ganglioside imaging. Salt-enhanced MALDI-TIMS and reactive-matrix approaches improve sensitivity, but differences in cationization, ion suppression, and adduct formation complicate direct comparison with conventional datasets [52,87].

### 5.6. Lipid Oxidation and Neuroinflammatory Microenvironments

Oxidative stress is a major component of AD pathology and can generate oxidized phospholipids, aldehyde-containing lipids, fragmented fatty acids, and lysophospholipids. These products are often low-abundance and chemically unstable and are therefore underrepresented in conventional MSI experiments [61].

MALDI-based cell fingerprinting of LPS-activated microglia revealed substantial inflammation-associated changes in phospholipid and sphingolipid profiles [90]. Although based on a cellular neuroinflammation model, the findings demonstrate that microglial activation is accompanied by a reproducible lipid phenotype that can contribute to plaque-associated MSI signals.

Multimodal MSI of LPS-induced neuroinflammation has identified accumulation of bioactive lipid species associated with inflammatory activation, incomplete fatty-acid oxidation, and oxidative stress [91]. Such models provide reference patterns for interpreting lipid signals located near activated microglia in AD tissue.

Recent work has directly linked specific plaque-associated lipids with microglial regulation. Microglia were shown to control accumulation of arachidonic-acid-containing bis(monoacylglycero)phosphate near amyloid pathology, whereas lysophospholipids were associated more strongly with astrocytic responses and oxidative stress [86]. This indicates that different lipid signals can mark distinct cellular processes within the same plaque microenvironment.

Bis(monoacylglycero)phosphate is enriched in late endosomes and lysosomes and may reflect enhanced phagocytosis, lysosomal stress, and degradation of membrane material by plaque-associated microglia. Its spatial accumulation provides a mechanistic link among microglial activity, lysosomal dysfunction, and altered lipid trafficking [86].

Oxidized phospholipids remain challenging to assign solely by accurate mass because oxidation produces many isomeric and isobaric products. Reactive derivatization, tandem MS, ion mobility, and LC–MS validation are required to distinguish true oxidized species from in-source fragments or preparation-induced oxidation [26,61].

### 5.7. Human AD Tissue and Experimental Models

Mouse models provide controlled age, genotype, environment, and disease stage, enabling longitudinal analysis that is impossible in human post-mortem tissue. They are therefore valuable for establishing temporal relationships among plaque formation, regional lipid remodeling, and neuroinflammation [76,79].

However, amyloid mouse models do not reproduce the complete human disease. They often develop extensive Aβ deposition without the full extent of neuronal loss, tau pathology, vascular disease, myelin degeneration, metabolic comorbidity, or decades-long aging observed in sporadic AD. Lipid signatures detected in APP/PS1, APPPS1, tgArcSwe, or related models should therefore not automatically be interpreted as universal human AD patterns [11,17].

This distinction is also relevant to familial and sporadic AD. Amyloid-overexpressing transgenic models predominantly reproduce genetically driven aspects of amyloid pathology and may more closely reflect selected mechanisms of familial AD than the multifactorial biology of sporadic late-onset disease. Sporadic AD is additionally shaped by aging, APOE genotype, vascular pathology, metabolic status, inflammatory history, and heterogeneous tau burden. Spatial lipid signatures derived from familial-AD-related models should therefore be validated in neuropathologically characterized sporadic human cohorts.

Human single-plaque imaging has confirmed several observations from mouse models, including ganglioside enrichment, local sulfatide depletion, and plaque-associated ceramide and phospholipid remodeling [75]. This cross-species correspondence strengthens the biological relevance of plaque lipid microenvironments.

Nevertheless, human tissue exhibits greater interindividual and regional heterogeneity. Post-mortem interval, APOE genotype, disease stage, sex, medication, vascular pathology, myelin content, and anatomical sampling can influence the measured lipidome. Integrated spatial multiomics and careful neuropathological annotation are needed to separate AD-specific changes from these covariates [77].

The human hippocampus requires particularly precise anatomical segmentation because its trisynaptic circuit contains molecularly distinct subfields. Histology-guided spatial lipidomics and proteomics have been developed to resolve these subregions and reduce dilution of localized signals [92]. Such workflows may be especially useful for studying early AD changes in vulnerable hippocampal circuits. Future studies should combine this subfield-level analysis with Aβ and tau staging to determine whether lipid remodeling follows amyloid burden, tau propagation, neuronal vulnerability, or distinct combinations of these processes.

Organoids, cultured microglia, and ex vivo brain slices provide intermediate experimental systems in which specific genetic or inflammatory mechanisms can be manipulated. However, their lipid composition, maturation state, cellular diversity, and myelination differ from adult human brain, and spatial findings require validation in intact tissue [51,90,93].

### 5.8. Biological Interpretation and Current Limitations

The most reproducible spatial findings in AD include plaque-associated accumulation of GM2 and GM3, local enrichment of selected ceramide-related and phosphatidylinositol species, depletion of several sulfatides, and broader region-dependent remodeling of glycerophospholipids and sphingolipids [15,44,69,70,75].

Other observations, including cholesterol accumulation, oxidized lipid distributions, lipid-droplet composition, and low-abundance signaling lipids, remain more dependent on specialized matrices, derivatization, metal-assisted ionization, or complementary MSI platforms [61,62,87,88]. These findings are promising but currently less comparable across laboratories.

The biological origin of an MSI signal cannot be determined from spatial co-localization alone. Lipids detected within a plaque may originate from amyloid-associated membranes, dystrophic neurites, extracellular vesicles, lysosomes, myelin debris, astrocytes, microglia, or preparation-induced fragments. Multimodal imaging, cell-type markers, tandem MS, and orthogonal lipidomics are required to assign cellular and molecular provenance [44,46,79].

Similarly, increased ion intensity does not necessarily represent increased concentration. Local matrix crystallization, salt content, tissue density, ion suppression, adduct formation, and extraction efficiency can differ between plaque and non-plaque regions. Internal standards and orthogonal quantification improve confidence but cannot fully reproduce the microchemical environment of an individual lesion [20,41].

**Figure 2:**
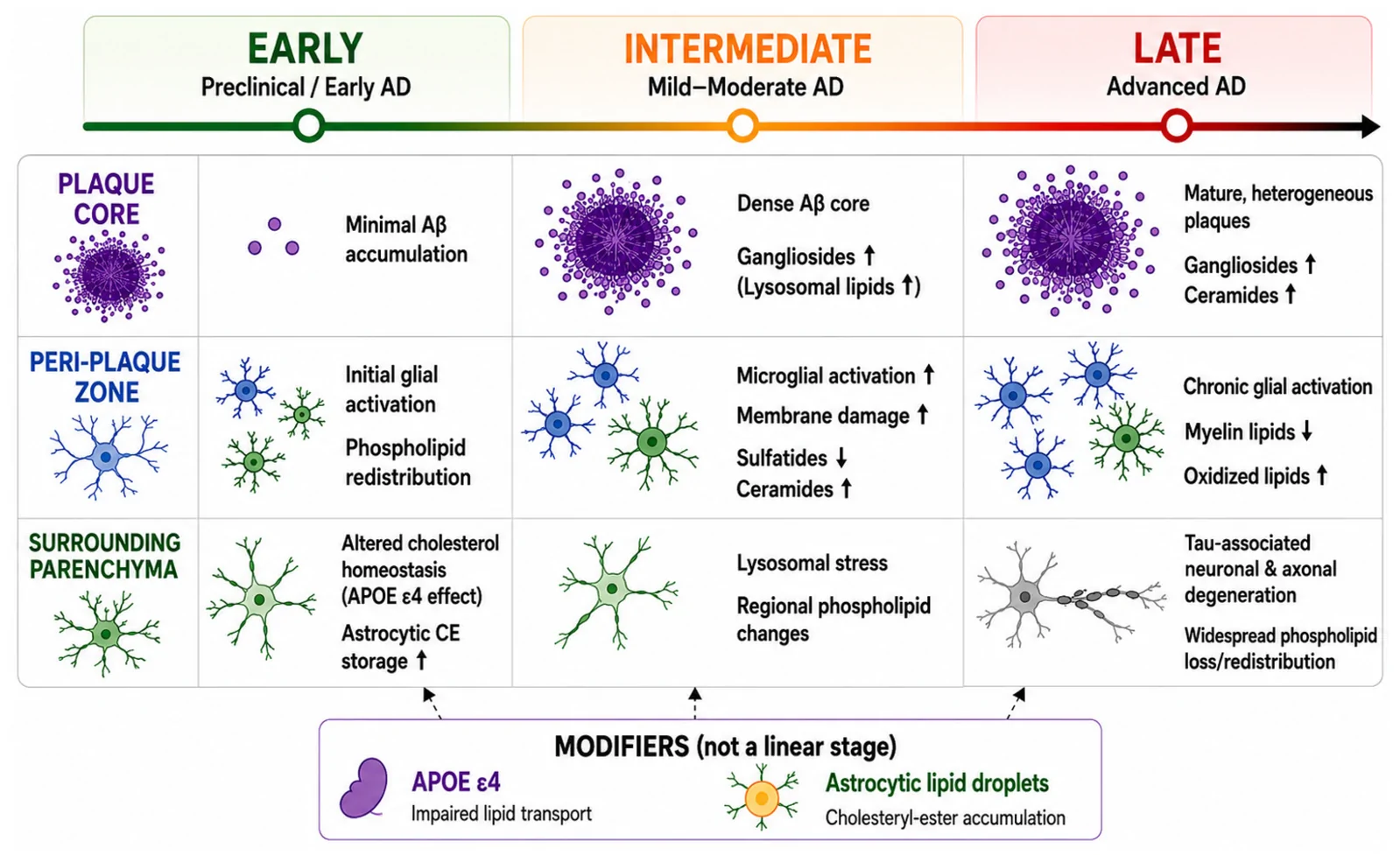
Proposed spatiotemporal evolution of lipid remodeling in Alzheimer’s disease.

The proposed temporal and spatial relationships among amyloid deposition, glial activation, lysosomal processing, myelin disruption, tau-associated degeneration, and lipid remodeling are summarized in Figure 2.

The schematic presents early, intermediate, and late phases of AD-associated lipid remodeling across the plaque core, peri-plaque zone, and surrounding parenchyma. Early changes involve altered membrane and cholesterol homeostasis, initial glial responses, and region-specific phospholipid redistribution. Intermediate stages are characterized by increasing amyloid-β deposition, peri-plaque membrane damage, ganglioside processing, lysosomal lipid accumulation, ceramide-related remodeling, and local sulfatide depletion. Later stages involve mature heterogeneous plaques, persistent microglial and astrocytic activation, myelin disruption, chronic oxidative stress, broader phospholipid loss or redistribution, and tau-associated neuronal and axonal degeneration. APOE ε4-dependent lipid transport and astrocytic cholesteryl-ester storage are represented as modifiers of these processes rather than as independent linear stages.

Overall, MALDI-MSI has shifted the interpretation of lipid dysregulation in AD from a model of global tissue imbalance toward one of anatomically and cellularly organized lipid pathology. The available evidence indicates that amyloid plaques act as focal sites of membrane degradation, glycosphingolipid processing, lysosomal stress, glial activation, and altered cholesterol and phospholipid trafficking.

However, the available evidence remains strongest for amyloid plaque-associated microenvironments and less developed for tau-associated lipid remodeling, APOE ε4-stratified spatial phenotypes, and differences between familial and sporadic AD. Future studies must therefore integrate high-spatial-resolution MSI with structural lipid identification, cell-type-resolved markers, longitudinal models, Aβ and tau neuropathological staging, APOE genotyping, and well-characterized human cohorts to determine which spatial lipid changes are causal, compensatory, or secondary to neurodegeneration [75,76,79,86].

## 6. Emerging Analytical Strategies in Alzheimer’s Disease Spatial Lipidomics

Conventional MALDI-MSI provides direct molecular maps of brain lipids, but its analytical depth is limited by ion suppression, incomplete ionization of neutral and low-abundance compounds, isobaric overlap, lipid isomerism, in-source fragmentation, and the progressive loss of sensitivity as pixel size decreases. Emerging approaches address these limitations by adding post-ionization, gas-phase separation, structure-selective fragmentation, reactive chemistry, multimodal co-registration, or computational annotation to the imaging workflow. The following sections are organized according to the principal analytical limitation addressed by each strategy: insufficient sensitivity, spectral overlap, incomplete structural identification, restricted chemical coverage, limited pathological context, and insufficient spatial or computational resolution [17,21,26].

These developments are particularly relevant to Alzheimer’s disease (AD), where meaningful lipid alterations may occur within individual amyloid plaques, narrow peri-plaque zones, dystrophic neurites, activated glial cells, lysosomal compartments, and specific membrane microdomains. Increased signal intensity alone is therefore insufficient; emerging methods must improve molecular specificity and spatial fidelity while remaining compatible with fragile human brain specimens and neuropathological validation [44,75,79].

### 6.1. MALDI-2 and Other Post-Ionization Strategies

MALDI-2 introduces a second laser that intercepts the expanding MALDI plume and post-ionizes neutral or weakly ionized molecules. This can increase sensitivity for lipid classes that are suppressed or inefficiently ionized during conventional MALDI, particularly when only small amounts of analyte are sampled at high spatial resolution [21,24].

Salviati et al. applied MALDI-2 to visualize endocannabinoids in mouse brain and obtained spatial information for low-abundance lipid mediators that are difficult to detect using conventional MALDI-MSI [94]. This capability is relevant to AD because endocannabinoid signaling, neuroinflammation, synaptic dysfunction, and glial responses are spatially heterogeneous and may involve compounds below the detection limits of standard workflows.

Post-ionization does not enhance all tissue ions equally. A machine-learning comparison of MALDI and MALDI-2 brain images demonstrated that some ion distributions are preserved, whereas others differ in intensity, localization, or detectability between the two modes [95]. MALDI-2 datasets should therefore not be interpreted simply as higher-sensitivity versions of conventional MALDI data.

The effect of MALDI-2 depends on matrix chemistry, desorption-laser fluence, post-ionization wavelength, laser alignment, plume density, delay time, gas pressure, and tissue composition. Regional differences in brain tissue may consequently influence post-ionization efficiency, creating class- and tissue-dependent enhancement rather than a constant multiplicative gain [21,24,95].

Plasma-based post-ionization provides an alternative strategy. Michael et al. coupled atmospheric-pressure MALDI with plasma post-ionization and trapped ion mobility spectrometry, increasing the detection of several lipid classes while simultaneously providing mobility separation [96]. This combination addresses sensitivity and spectral congestion within one multidimensional experiment.

Infrared MALDI coupled with dielectric-barrier-discharge plasma post-ionization has also improved the detection of nonpolar metabolites, including sterol- and neutral-lipid-related compounds [97]. Such approaches may broaden spatial analysis beyond the phospholipids and glycosphingolipids that dominate conventional AD lipid-MSI datasets.

Single-photon-induced post-ionization represents another route for increasing ion yield. Bookmeyer et al. demonstrated substantial enhancement of lipid classes that ordinarily experience strong ion suppression during MALDI-MSI [98]. The availability of several post-ionization mechanisms indicates that the optimal approach may depend on analyte ionization energy, polarity, matrix system, and instrument geometry.

Post-ionization introduces additional requirements for quality control. Enhancement factors should be characterized by lipid class and anatomical region, while comparisons with conventional MALDI, standards, and orthogonal LC–MS/MS are needed to determine whether newly observed signals represent intact lipids, alternative adducts, or post-ionization products. Thus, post-ionization primarily addresses sensitivity, but it does not by itself resolve spectral overlap or establish lipid structure, which motivates the use of ion mobility and structure-selective methods [21,95,96,97,98].

### 6.2. Ion Mobility and Separation of Isobaric Lipids

Ion mobility–mass spectrometry separates ions according to their gas-phase mobility, which depends on charge, size, shape, conformation, and interactions with the drift gas. When incorporated into MSI, this separation reduces spectral overlap and provides an additional molecular descriptor in the form of drift time or collision cross section [17,21].

Trapped ion mobility spectrometry has already demonstrated its value in AD research. TIMS-MSI revealed that structurally distinct amyloid plaques are associated with different lipid profiles and enabled the extraction of molecular features that would remain unresolved in mass-only images [71]. Ion mobility therefore supports both chemical and pathological stratification of plaque heterogeneity.

Multimodal plaque- and region-specific imaging has extended this approach by integrating MALDI-2, TIMS, fluorescence microscopy, and computational segmentation [79]. The combination increases sensitivity while distinguishing mobility-resolved lipid features associated with defined plaques or anatomical regions.

Other mobility platforms can provide complementary separation. DESI cyclic ion mobility MSI has resolved and imaged individual lipid isobars in injured brain tissue, demonstrating the potential of repeated mobility separation for complex neurological samples [99]. Although developed outside AD, the approach is directly relevant to plaque-associated phospholipids that share nominal or exact masses.

Collision cross section values can strengthen molecular annotation when compared with validated standards or curated databases. However, CCS values depend on the ion type, adduct, charge state, calibration method, gas composition, and mobility platform. Cross-platform CCS comparisons should therefore use harmonized calibration procedures and explicit tolerances rather than treating CCS as an absolute structural identifier.

Ion mobility cannot resolve every lipid isomer. Closely related double-bond positional, geometric, and sn-positional isomers may retain similar collision cross sections, while different conformers of one lipid can broaden or split mobility peaks. Mobility separation should therefore be combined with tandem MS or structure-selective reactions when detailed lipid structure is biologically important. Accordingly, ion mobility narrows the range of plausible assignments but does not eliminate the need for targeted structural lipidomics [21,79].

### 6.3. Imaging of Isobaric and Isomeric Lipids

Lipid annotation based solely on accurate *m*/*z* frequently stops at the sum-composition level. Multiple molecular structures may share the same total carbon number and number of double bonds while differing in fatty-acyl chains, sn-position, double-bond location, double-bond geometry, ether linkage, or headgroup composition. These distinctions influence membrane organization, enzymatic metabolism, oxidation, and signaling [100].

A broad review of isomer-selective MSI methods identified ion mobility, ozone-induced dissociation, ultraviolet photodissociation, electron-based fragmentation, Paternò–Büchi chemistry, and gas-phase ion/ion reactions as complementary approaches for visualizing lipid isomers in biological tissues [100]. No single strategy currently resolves all relevant levels of lipid structure.

Gas-phase charge inversion has been used to distinguish isobaric phosphatidylcholines in MSI. Specker and Prentice converted lipid cations into informative anionic complexes, enabling class-selective reactions and spatial differentiation of ions that could not be separated from their precursor *m*/*z* values alone [101].

The addition of infrared multiphoton dissociation to charge-inversion imaging enabled relative quantification and structural discrimination of lipid isomers at individual imaging pixels [102]. This approach demonstrates that gas-phase chemistry can provide both spatial and structural information without requiring on-tissue derivatization.

Selective Schiff-base-forming ion/ion reactions have similarly distinguished isobaric lipid classes and revealed their separate tissue distributions [103]. These methods are particularly valuable when phospholipids of different classes generate overlapping ions but undergo different reactions with a structure-selective reagent.

Such gas-phase approaches preserve the tissue preparation workflow but require specialized instrumentation, reaction-control capabilities, and sufficiently intense precursor ions. Their throughput may also be lower than that of full-scan MSI because additional reaction and fragmentation steps are performed at many pixels. These approaches establish lipid class or structural differences that remain hidden in conventional MS1 images, whereas localization of double bonds and sn-positions requires further structure-selective analysis [101,102,103].

### 6.4. Double-Bond and sn-Position-Resolved Structural Lipidomics

Double-bond location is biologically important because it influences membrane fluidity, lipid oxidation, receptor interactions, and enzymatic conversion into signaling mediators. Conventional collision-induced dissociation often identifies lipid headgroups and fatty-acyl composition but does not reliably localize carbon–carbon double bonds [100].

On-tissue 3-acetylpyridine Paternò–Büchi derivatization has enabled high-coverage imaging of double-bond-location isomers from glycerophospholipids, fatty acids, and neutral lipids [104]. Photochemical addition across the double bond generates diagnostic fragment pairs that distinguish isomers sharing the same precursor mass.

Paternò–Büchi workflows require careful control of illumination, reaction time, reagent deposition, crystal formation, and tissue wetting. Incomplete or spatially nonuniform reaction can create apparent regional differences in isomer abundance, while side products increase spectral complexity [104].

Ozone-induced dissociation provides another route to double-bond localization. Ozone reacts selectively with carbon–carbon double bonds and produces position-specific fragments. The LipidOz computational workflow has automated the interpretation of OzID spectra and improved assignment of double-bond positions in complex lipidomic datasets [105].

The translation of OzID into imaging remains constrained by reaction time, ion abundance, instrument architecture, and the number of candidate precursors interrogated. Nevertheless, automated data interpretation is an important step toward applying structure-resolved lipid analysis across large spatial datasets [105].

AD-associated phospholipid remodeling can also be specific to the sn-position of fatty-acyl chains. High-resolution ion-mobility-enabled lipidomics resolved spatial and temporal changes in glycerophospholipid sn-isomers in an AD mouse model, revealing molecular patterns that would be obscured by sum-composition annotation [106].

The biological significance of sn-position arises from phospholipase selectivity, fatty-acid release, membrane curvature, and the generation of lipid mediators. Two isomers with identical fatty-acyl composition can therefore follow different metabolic pathways and show different regional changes during aging or amyloid progression [106].

Cyclic ion mobility imaging has recently been applied to resolve sn-positional isomers of docosahexaenoic-acid-containing phospholipids in mouse brain [107]. DHA-containing phospholipids are highly relevant to neuronal membranes and oxidative vulnerability, making this analytical direction particularly important for future AD studies.

Structural assignments remain limited by the availability of authentic lipid standards. Many double-bond and sn-positional isomers are not commercially available, and predicted fragments or CCS values cannot fully replace empirical validation. Reports should clearly distinguish direct structural confirmation from tentative isomer assignment. When the analytical objective instead concerns poorly ionizing lipid classes rather than isomeric structure, reactive matrices and on-tissue derivatization provide a complementary solution [100,106,107].

### 6.5. Reactive Matrices and On-Tissue Chemical Derivatization

Reactive matrices and on-tissue derivatization expand MSI toward compounds with poor conventional ionization, including cholesterol, glycerides, short-chain fatty acids, free fatty acids, volatile metabolites, and oxidized lipids. These strategies introduce a chargeable group, improve desorption, shift the analyte away from matrix interference, or generate structure-specific fragments [25,61,87].

Recent methodological reviews have documented rapid expansion of on-tissue derivatization strategies for MALDI-MSI, including reactions directed toward carboxyl, carbonyl, hydroxyl, amine, and unsaturated functional groups [108]. The principal benefit is access to chemical classes that are underrepresented in standard positive- or negative-ion lipid imaging.

Isotope-coded on-tissue derivatization has enabled quantitative imaging of short-chain fatty acids in biological tissues [109]. Isotopic labeling can correct part of the variability in reaction yield and ionization, although true quantitative imaging still requires calibration, validation of spatial uniformity, and control of endogenous matrix effects.

Vacuum-promoted derivatization has been used to map glycerides, which are difficult to ionize because of their neutral character [110]. Vacuum conditions can enhance reagent penetration and reaction efficiency while reducing some solvent-related spreading, but the procedure still requires direct experimental assessment of lipid delocalization.

Reactive-matrix approaches have already improved cholesterol imaging in brain tissue [62,87], while derivatization with hydrazide reagents has increased detection of selected oxidized phospholipids [61]. These methods may reveal sterol and oxidative-lipid pathology that is absent from conventional phospholipid-centered datasets.

The principal limitation of on-tissue chemistry is that the reaction itself becomes part of the spatial measurement. Reagent purity, deposition homogeneity, solvent composition, reaction kinetics, humidity, temperature, tissue penetration, and side reactions can all influence image intensity. Appropriate controls include derivatized standards, tissue blanks, adjacent untreated sections, tandem MS, and orthogonal analysis. Chemical expansion of molecular coverage therefore increases the need for pathological co-registration and multimodal validation, particularly when reactive products are localized to small plaque-associated regions [61,108,109,110].

### 6.6. Multimodal and Spatial Multiomic Workflows

AD pathology involves interactions among lipids, Aβ peptides, tau, glycans, inflammatory proteins, cell-type markers, and tissue architecture. Spatial lipid maps therefore gain biological meaning when integrated with histology, immunohistochemistry, fluorescence microscopy, proteomics, glycomics, or complementary imaging modalities [44,46,72].

Sequential spatial multiomics has enabled analysis of lipids, N-glycans, and tryptic peptides from the same FFPE tissue section [111]. The same-section design reduces anatomical mismatch but requires a carefully ordered workflow because delipidation, enzymatic digestion, antigen retrieval, and washing can affect downstream molecular layers.

Spatial multiomics reviews emphasize that integrating molecular modalities does not automatically produce biologically coherent data [112]. Each modality has distinct spatial resolution, sensitivity, dynamic range, normalization requirements, and susceptibility to tissue-processing artifacts.

AD-specific spatial multiomic frameworks have similarly highlighted the need to connect lipid distributions with amyloid, tau, glial, vascular, and neuronal markers [113]. These integrations can distinguish plaque-associated lipid accumulation from regional background and help assign signals to specific pathological processes.

Sequential lipid–glycan–peptide imaging has been demonstrated in fresh-frozen AD-model brain [27], while post-mortem human AD studies have integrated intact MSI, on-tissue protein digestion, lipid imaging, and LC–MS/MS [77]. These approaches maximize information from scarce specimens but increase analytical complexity.

The same-section strategy provides direct co-localization, whereas adjacent-section analysis avoids chemical interference between modalities. The appropriate design depends on lesion size, tissue availability, section-to-section similarity, and the compatibility of analytical reagents [44,46].

Recent sequential MALDI-MSI workflows in tumor tissue have further demonstrated that lipid, glycan, and peptide signatures can be integrated with histopathology in one spatial analytical sequence [114]. Although developed outside neurodegeneration, these workflows provide technical models for expanding AD tissue analysis.

Multimodal registration must be reported quantitatively. Rigid alignment may be sufficient for minimally processed sections, but matrix removal, washing, digestion, staining, and mounting can cause nonlinear tissue deformation. Landmark-based or deformable registration should therefore include an estimate of spatial error, especially when structures approach the pixel size. After molecular identity and pathological context have been established, three-dimensional and cellular-scale approaches can define how far these changes extend through tissue and among individual cell populations [44,72].

### 6.7. Three-Dimensional, Single-Cell, and Subcellular Imaging

Three-dimensional MSI reconstructs molecular distributions from serial tissue sections and can reveal whether lipid abnormalities extend through an anatomical volume or are restricted to individual planes. In AD-model brain, 3D integration of lipid MSI and immunohistochemistry has demonstrated region- and depth-dependent molecular changes [72].

The reliability of 3D reconstruction depends on consistent section thickness, orientation, matrix deposition, acquisition, normalization, and registration. Section loss or variable ionization can produce artificial discontinuities, while differences in anatomical depth may be mistaken for temporal or pathological gradients [72].

Single-cell MSI aims to resolve lipid heterogeneity among neurons, microglia, astrocytes, oligodendrocytes, and other cells. Reviews of single-cell and subcellular lipid MSI emphasize that spatial resolution alone is not sufficient: sensitivity, sampling depth, matrix-crystal size, analyte migration, and cell identification must also be addressed [115].

Dual-polarity ionization combined with ion mobility has enabled in situ profiling of lipid diversity in individual cells and mouse brain [58]. The complementary polarities improve lipid-class coverage, while ion mobility reduces overlap among species detected from small cellular areas.

Transmission-geometry ambient laser desorption combined with plasma ionization has achieved subcellular imaging of lipids and nucleotides at micrometre-scale pixel sizes [116]. Transmission geometry reduces constraints associated with conventional laser focusing and may improve access to small cellular structures.

Ultrahigh-resolution 21 T MALDI-FTICR has also been applied to single-cell and subcellular analysis, coupling high mass resolving power with post-MSI immunocytochemical identification of cell types [117]. This approach addresses spectral specificity but remains limited by acquisition time, instrument availability, and the low analyte amount within individual cells.

MALDI-MSI studies of cultured cells have shown that chemical heterogeneity can be detected even among cells grown under nominally identical conditions [118]. This observation is important for AD because glial and neuronal responses to plaques may vary between neighboring cells.

High-resolution acquisition involves a fundamental sensitivity trade-off. As pixel area decreases, less analyte is sampled, increasing the importance of MALDI-2, efficient matrix application, high-transmission instruments, oversampling, and optimized data processing [22,23,24].

MALDI-2-enabled oversampling has improved metabolite and lipid imaging at single-cell resolution by combining a small effective sampling interval with post-ionization sensitivity enhancement [24]. However, oversampling can introduce spatial convolution if successive laser positions interrogate partially depleted or overlapping material.

Cellular identity should ideally be established using histology, fluorescence, immunolabeling, or morphology rather than inferred solely from lipid clusters. Spatial co-registration between MSI and cell markers is necessary before assigning a molecular signature to a defined neural or glial population. The resulting high-dimensional datasets require computational methods that retain the relationships among spatial coordinates, molecular structure, cellular identity, and pathological annotation [44,115,116,117,118].

### 6.8. Computational Analysis, Co-Registration, and Molecular Annotation

Emerging MSI platforms generate multidimensional datasets containing spatial coordinates, accurate mass, intensity, polarity, ion mobility, fragmentation spectra, derivatization products, and multimodal image layers. Reliable interpretation requires processing pipelines that preserve these relationships rather than reducing the experiment to independent single-ion images [21,79].

Data preprocessing includes baseline correction, denoising, recalibration, peak alignment, normalization, isotope grouping, adduct recognition, matrix-peak removal, and correction for batch effects. Each step can alter downstream segmentation and statistical significance and should therefore be documented with sufficient detail for reproduction [20,21].

In-source fragmentation creates a particular annotation problem in lipid MALDI-MSI. The rMSIfragment workflow was developed to identify spatially correlated precursor–fragment relationships and reduce false lipid annotations caused by source-generated fragments [119]. This is highly relevant when fragment ions resemble plausible endogenous fatty acids, diacylglycerols, or headgroup-loss products.

Annotation-scoring systems can make identification confidence more transparent. The metabolite annotation confidence score integrates mass accuracy, spectral evidence, and spatial behavior to rank MSI assignments [120]. Such scoring does not replace structural validation but helps distinguish strongly supported annotations from tentative database matches.

Benchmark datasets are required to compare annotation tools objectively. A sulfatide-centered ultrahigh-resolution MALDI imaging dataset has been developed for evaluating MS1-based lipid annotation pipelines and testing their reproducibility [121]. Similar reference datasets for gangliosides, phosphoinositides, oxidized lipids, and plaque-associated compounds would benefit AD research.

Machine learning can support tissue segmentation, plaque detection, feature prioritization, classification, and integration of MALDI and MALDI-2 images [79,95]. However, high dimensionality and limited biological sample numbers create a substantial risk of overfitting.

Machine-learning models should be evaluated using independent biological specimens rather than random pixels from the same tissue section. Pixel-level splitting can produce artificially high accuracy because neighboring pixels are spatially correlated and share technical variation.

Histological co-registration introduces another computational uncertainty. Differences in section deformation, pixel size, orientation, and tissue damage should be incorporated into the interpretation of co-localization. A lipid signal should not be described as plaque-localized when registration error is comparable to the width of the peri-plaque region [44,72].

Lipid nomenclature should reflect the level of structural evidence. An *m*/*z* match may support only a lipid class and sum composition, whereas fatty-acyl composition, double-bond position, and sn-position require progressively stronger evidence from MS/MS, mobility, derivatization, authentic standards, or orthogonal LC–MS/MS. These computational and reporting requirements determine whether the additional dimensions generated by emerging technologies improve biological interpretation or merely increase analytical complexity [100,101,102,103,104,105,106,107].

### 6.9. Current Limitations and Analytical Priorities

The increased dimensionality of emerging MSI does not automatically resolve all analytical ambiguity. MALDI-2 improves sensitivity but may produce class-dependent enhancement; ion mobility separates some but not all isomers; derivatization expands chemical coverage but introduces reaction variability; and multimodal analysis increases biological context while complicating tissue handling and registration [95,96,108,112].

Throughput remains a major limitation. High-resolution imaging, multiple polarities, ion mobility, pixel-resolved MS/MS, and 3D acquisition generate long measurement times and large datasets. This constrains cohort size and may make a technically sophisticated study less statistically representative.

Quantitative interpretation also remains difficult. Post-ionization enhancement, mobility transmission, derivatization yield, and fragmentation efficiency can vary among lipid classes and tissue regions. Internal standards and calibration models must therefore be adapted to the complete multidimensional workflow rather than borrowed from conventional MALDI-MSI [41].

Interlaboratory reproducibility will require shared reference materials, standardized reporting, publicly available raw data, common data formats, and benchmark datasets. Without these elements, additional analytical dimensions may increase laboratory-specific variation instead of improving cross-study comparability [21,121].

For AD research, the most important priority is not maximum technical complexity but alignment between technology and biological question. Plaque-scale ganglioside imaging, structural analysis of phospholipid isomers, cholesterol mapping, glial lipid-droplet analysis, and 3D regional mapping require different combinations of sensitivity, spatial resolution, structural specificity, and multimodal validation [62,75,79].

These strategies should be selected according to the analytical limitation that prevents a specific AD-related question from being answered. Post-ionization is most appropriate when sensitivity is limiting; ion mobility and structure-selective fragmentation are required when isobaric or isomeric ambiguity dominates; derivatization is valuable for poorly ionizing lipid classes; multimodal workflows establish pathological and cellular context; and three-dimensional or single-cell imaging addresses spatial heterogeneity. This decision-based framework avoids presenting technical complexity as an objective in itself.

Overall, emerging analytical strategies are moving AD spatial lipidomics beyond descriptive maps of nominal *m*/*z* values toward multidimensional molecular histology. Their successful application will depend on combining increased sensitivity and structural detail with rigorous validation, transparent annotation, quantitative controls, accurate pathology co-registration, and biologically representative cohorts [21,75,79,100].

## 7. Translational Challenges, Standardization, and Clinical Potential

MALDI mass spectrometry imaging has become an established research platform for mapping lipids in neurological tissue, but its transition into translational Alzheimer’s disease research and clinical neuropathology remains incomplete. The principal barriers are not limited to instrumental sensitivity. They include preanalytical variability, incomplete quantitative validation, inconsistent molecular annotation, limited cohort sizes, nonuniform reporting, and insufficient evidence that results can be reproduced across laboratories, instruments, and tissue collections [17,21].

For AD applications, these challenges are amplified by the heterogeneity of post-mortem brain tissue. Lipid distributions depend on anatomical region, grey- and white-matter content, disease stage, plaque density, vascular pathology, glial activation, genotype, and post-mortem handling. A clinically interpretable spatial lipid signature must therefore remain robust after accounting for both biological and technical sources of variation [65,75,77].

### 7.1. Preanalytical Standardization

The analytical result of lipid MALDI-MSI is strongly influenced by events occurring before data acquisition. Tissue collection, post-mortem interval, freezing rate, storage duration, freeze–thaw exposure, cryosectioning, mounting, drying, washing, matrix application, and environmental humidity can change lipid extraction, oxidation, adduct formation, and tissue morphology [27,41].

Integrated MALDI-MSI–LC–MS/MS workflows have demonstrated that apparently minor changes in slide type, tissue handling, extraction, and internal-standard application can affect both imaging and downstream quantitative lipidomics [122]. Preanalytical procedures should therefore be treated as controlled experimental variables rather than routine laboratory details.

A quantitative lipid MALDI-MSI workflow incorporating an LC–MS-based quality-control pipeline further illustrates the need to monitor sample preparation, signal stability, calibration performance, and agreement between imaging and extract-based measurements [123]. Such orthogonal quality control is particularly important when regional intensity differences are interpreted as biological concentration changes.

Tissue-section thickness is another underappreciated source of variability. A systematic investigation demonstrated that section thickness influences signal intensity, molecular coverage, image quality, and the balance between analyte abundance and matrix-related effects [124].

In AD cohorts, anatomical sampling must also be standardized. Sections described broadly as “cortex” or “hippocampus” may include different cortical layers, white-matter proportions, subfields, vascular structures, and plaque burdens. Sampling protocols should specify anatomical landmarks, laterality, section plane, distance from reference structures, and the relationship between the imaged section and the neuropathological evaluation [65,75].

Fresh-frozen tissue currently offers the broadest access to endogenous lipids, but formalin-fixed paraffin-embedded material is much more abundant in clinical archives. FFPE lipid analysis remains affected by solvent extraction, chemical modification, paraffin removal, and prolonged storage. Consequently, fresh-frozen and FFPE datasets should not be combined without method-specific validation and explicit recognition of their different detectable lipidomes [27,111].

### 7.2. Analytical Reproducibility and Interlaboratory Comparability

Within-laboratory repeatability does not guarantee interlaboratory reproducibility. Matrix deposition, crystal size, laser fluence, raster size, detector response, polarity, ion optics, mass analyzer, calibration strategy, and data-processing software can all alter the number, intensity, and apparent localization of lipid ions [20,21].

A multicenter evaluation of MALDI-MSI tissue classification showed that reproducible classification is achievable across sites, but only when sample preparation, acquisition, preprocessing, and statistical analysis are sufficiently controlled [125]. This is directly relevant to AD because disease classification based on spatial lipid profiles will require performance beyond a single instrument or laboratory.

Reporting standards are central to reproducibility. The SMART reporting standard was developed to improve documentation of sample preparation, acquisition parameters, data processing, image generation, statistical analysis, and data availability in MSI studies [126]. Adoption of such structured reporting would allow readers to determine whether differences between AD studies reflect biology or incompatible analytical workflows.

Clinical diagnostic perspectives on MSI similarly emphasize that reproducibility, validation, reference procedures, and transparent analytical performance are prerequisites for routine implementation [127]. An imaging result cannot be considered clinically actionable solely because it distinguishes groups in a discovery cohort.

MSI studies directed toward personalized medicine have demonstrated the value of combining molecular images with histopathology and patient-level information, while also showing that insufficient method validation limits direct clinical adoption [128]. For AD, the corresponding translational objective would be to connect spatial lipid patterns with neuropathological stage, genotype, disease duration, cognitive phenotype, and treatment exposure.

Reviews of state-of-the-art biomedical MSI applications identify multicenter studies, common reference materials, harmonized preprocessing, and external validation as major requirements for translation [129].

### 7.3. Quantitative and Semi-Quantitative Lipid MSI

Most lipid MALDI-MSI studies report normalized ion intensities rather than absolute concentrations. These values are affected by local ion suppression, salt content, matrix crystallization, lipid class, adduct competition, detector response, and tissue composition. Relative images are highly informative spatially but should not automatically be interpreted as quantitative concentration maps [20,41].

A multiclass internal-standard mixture has enabled omics-scale quantitative MSI of nearly 200 lipids in brain tissue across different MALDI modes and mass analyzers [41]. This work demonstrated the feasibility of broad quantitative imaging but also showed that accuracy depends on the structural similarity between analyte and standard, the lipid class, ion type, and calibration model.

The MALDI-MSI–LC–MS/MS workflow of Hendriks et al. showed that internal standards strongly influence quantitative agreement between imaging and extract-based lipidomics [122]. Standards deposited onto the tissue surface do not fully reproduce endogenous extraction, cellular localization, or ion suppression, but they provide essential correction for analytical variability.

The LC–MS-based quality-control strategy proposed by Hormann and Heiles offers a framework for verifying lipid identities and evaluating the quantitative performance of MALDI-MSI [123]. Agreement between imaging and LC–MS should be assessed at the level of individual lipid species rather than inferred from class-wide correlations.

Quantitative MSI surveys in pharmaceutical development indicate that validation should address selectivity, calibration range, accuracy, precision, stability, recovery, matrix effects, spatial reproducibility, and the influence of tissue heterogeneity [130]. Although these principles were developed largely for drug imaging, they are applicable to endogenous lipid measurements.

For AD studies, full absolute quantification may not always be necessary. Semi-quantitative imaging can be sufficient when the objective is to compare plaque cores, peri-plaque regions, or defined anatomical compartments. However, the analytical unit, normalization strategy, internal standard, segmentation method, and biological replication must be specified.

### 7.4. Molecular Identification and Reporting Standards

Clinical interpretation requires greater identification confidence than exploratory spatial profiling. Accurate mass alone generally supports only a tentative assignment, particularly in lipid-rich brain tissue containing isotopes, adducts, fragments, isobars, and structural isomers [100,119].

Diagnostic and toxicologic pathology applications of MSI increasingly combine imaging with histology, targeted MS/MS, orthogonal assays, and validated molecular markers [131]. These practices provide a useful model for AD, where the distinction between a plausible lipid assignment and a confirmed molecular species can change the biological interpretation.

LipidSpace was developed to support exploration, reanalysis, and quality control of large lipidomics datasets [132]. Although not restricted to MSI, such tools are valuable for identifying outliers, evaluating batch structure, comparing lipid profiles, and detecting inconsistencies before spatial interpretation.

Unified structural annotation frameworks can integrate full-scan LC–MS and MSI data, improving the transfer of molecular evidence between chromatographic and imaging experiments [133]. In AD research, this supports a workflow in which MSI provides localization while LC–MS/MS supplies higher-confidence structural evidence.

High-throughput biochemical mapping of brain tissue using deep learning has demonstrated that computational reconstruction and multiscale acquisition can increase spatial coverage and analytical throughput [134]. However, reconstructed images and predicted molecular distributions should remain distinguishable from directly measured data.

Evaluations of software for ion-mobility lipidomics show that annotation results depend on database coverage, adduct assumptions, CCS tolerances, scoring rules, and the quality of reference values [135]. Ion mobility adds valuable evidence, but a CCS match alone does not establish double-bond or sn-position identity.

Mobility-modulated sequential dissociation has enabled structurally informative lipid analysis while maintaining imaging capability [136]. Such strategies may reduce ambiguity in AD datasets, but the resulting structural claims should be matched to the actual diagnostic fragments obtained.

Recommendations for accurate lipid annotation and semi-absolute quantification emphasize appropriate extraction, internal standards, chromatographic separation, MS/MS evidence, and nomenclature reflecting the level of certainty [137]. These principles should also guide reporting of MSI-based lipid identities.

A lipid atlas developed using an annotation-focused workflow further demonstrated the importance of reference spectra acquired under multiple collision energies and explicit confidence levels [138]. Brain spatial lipidomics would benefit from comparable reference resources for gangliosides, sulfatides, phosphoinositides, oxidized lipids, and sterols.

Automated annotation tools such as MetaboAnnotatoR can improve consistency in large untargeted datasets, but automated results still require chemical plausibility checks and manual review of high-priority biomarkers [139]. Database matching should therefore be considered the beginning, not the end, of molecular identification.

Computational reviews of MSI emphasize that confident annotation, segmentation, co-registration, and pathway analysis depend on transparent preprocessing and validated statistical procedures [140]. For AD, computational sophistication cannot compensate for weak structural evidence or poorly controlled neuropathology.

### 7.5. Cohort Design and Neuropathological Annotation

Many AD MSI studies remain based on small numbers of brains or experimental animals. High-dimensional datasets containing thousands of pixels are not substitutes for independent biological replicates. Pixels from one section are spatially correlated and cannot be treated as independent patients.

Human cohorts should be balanced or statistically adjusted for age, sex, APOE genotype, post-mortem interval, brain bank, tissue pH, disease duration, medication, metabolic disease, vascular pathology, and cause of death. Neuropathological characterization should include Braak stage, CERAD score, Thal phase, Lewy body pathology, cerebral amyloid angiopathy, and relevant white-matter abnormalities where available.

The imaged region should be annotated at both anatomical and pathological levels. Region-wide analyses can detect broad lipid remodeling, while plaque-level segmentation can reveal highly localized effects. These levels should not be combined without hierarchical statistical models because multiple plaques from the same brain are not independent specimens [75].

Discovery and validation cohorts should be separated at the patient level. Cross-validation based on randomly divided pixels or spectra from the same tissue section will overestimate generalizability. External validation should ideally involve a different brain bank, instrument, operator, and acquisition batch.

Harmonization studies in high-resolution FT-ICR mass spectrometry demonstrate that instrument settings and acquisition parameters can be aligned across laboratories to improve comparability [141]. Comparable harmonization of MALDI-MSI platforms will be necessary before spatial lipid signatures can be transferred between centers.

Multicenter optimization of clinical mass-spectrometry workflows has further shown that standardized protocols, shared quality-control materials, and coordinated data analysis can reduce site-specific variation [142]. These principles are directly transferable to AD tissue-imaging consortia.

### 7.6. Clinical and Translational Applications

The most immediate clinical role of lipid MALDI-MSI in AD is likely to be molecular neuropathology rather than ante-mortem diagnosis. MSI can complement histology by identifying plaque-associated lipids, distinguishing molecularly different lesions, mapping regional vulnerability, and connecting protein pathology with local lipid metabolism [17,75].

MALDI-MSI has already been evaluated as a molecular histopathology tool in epilepsy surgery, where molecular profiles were compared with conventional neuropathological assessment [143]. This demonstrates that neurological tissue can be incorporated into clinically oriented MSI workflows, although AD presents different requirements because tissue is usually obtained post mortem.

MSI applications in neurology indicate potential roles in disease-mechanism studies, biomarker discovery, drug distribution, tissue classification, and pathological stratification [144]. For AD, clinically useful applications may include identifying molecular subtypes, evaluating plaque microenvironments, stratifying archived tissue, and mapping treatment-related changes in experimental studies.

Spatial lipid signatures may also support therapeutic development. Imaging can determine whether a drug reaches vulnerable brain regions, whether treatment modifies plaque-associated lipids, and whether biochemical effects occur in neurons, glia, white matter, or vascular compartments. Such pharmacodynamic mapping may be valuable even when the technique is not used as a clinical diagnostic test.

Another translational application is retrospective analysis of biobank material. Well-characterized brain collections can connect lipid distributions with longitudinal clinical data, genetics, neuropathology, and other omics layers. This requires harmonized metadata and careful control of differences in collection and storage.

### 7.7. Barriers to Routine Clinical Implementation

Routine clinical laboratories require methods with defined turnaround time, analytical robustness, quality-control acceptance criteria, trained personnel, secure data handling, and interpretable reports. Current lipid MALDI-MSI workflows often involve long acquisition times, complex preprocessing, manual annotation, and specialist-dependent interpretation [127,130].

Instrument cost and platform diversity are additional barriers. Results obtained by MALDI-TOF, Orbitrap, FT-ICR, timsTOF, MALDI-2, and ambient MSI cannot always be transferred directly because the platforms differ in sensitivity, resolving power, fragmentation, mobility separation, speed, and adduct behavior [21].

The absence of universally accepted reference ranges also limits clinical interpretation. Brain lipid composition varies strongly among regions and cell types, and a single “normal” value is unlikely to be appropriate. Reference datasets must therefore be anatomically resolved and stratified by relevant demographic and technical variables.

Regulatory implementation would require a clearly defined intended use. A method developed for exploratory plaque imaging cannot be validated in the same way as a test intended to classify patients or guide treatment. The analytical endpoint, decision threshold, error tolerance, and clinical consequence must be established before formal validation [130].

Data storage and computational traceability must also be addressed. Raw MSI datasets are large, and downstream results depend on numerous processing choices. Clinical use would require version-controlled software, audit trails, locked analysis pipelines, and preservation of raw and processed data.

### 7.8. Priorities for Translation

Translation should prioritize five coordinated requirements: standardized preanalytics, multicenter reproducibility, quantitative and structural validation, transparent reporting, and neuropathologically informed cohort design [41,75,77,122,123,124,125,126,132,133,134,135,136,137,138,139,140,141,142]. These priorities are interdependent and should be implemented as a unified translational framework rather than as separate technical objectives.

The major translational barriers and corresponding corrective actions are summarized in Table 4.

The translational development of lipid MALDI-MSI in AD will likely proceed through research-grade molecular neuropathology, multicenter validation, and therapeutic-response studies before routine diagnostic implementation. The technique offers a unique capacity to connect molecular composition with anatomical and pathological context, but clinical value will depend on standardization, quantitative credibility, structural identification, external validation, and demonstrable improvement over existing neuropathological methods [127,128,129,130,131].

Accordingly, the near-term translational value of lipid MALDI-MSI lies in reproducible molecular neuropathology and treatment-response mapping rather than routine ante-mortem diagnosis. Clinical implementation will require evidence that spatial lipid signatures add reproducible information beyond established neuropathological assessment and remain valid across independent cohorts, laboratories, and analytical platforms [125,126,127,128,129,130,131,141,142].

## 8. Conclusions and Future Perspectives

The principal consensus emerging from current spatial lipidomic research is that lipid pathology in AD is anatomically organized rather than uniformly distributed. The most reproducible findings include plaque-associated enrichment of GM2 and GM3 gangliosides, selected ceramide-related and phosphatidylinositol species, lysosomal lipids, and other membrane-derived phospholipids, together with local or regional depletion of several sulfatides [15,44,69,70,75]. These patterns support a model in which amyloid plaques represent heterogeneous lipid–cellular niches shaped by membrane degradation, glycosphingolipid processing, lysosomal stress, glial activation, and disruption of myelin-associated lipid homeostasis. Their composition varies with plaque morphology, anatomical location, cellular response, and disease stage, and therefore cannot be reduced to a single universal plaque-associated lipid signature [71,72,75,79].

At the same time, the available evidence remains uneven. Spatial relationships between lipids and amyloid pathology are considerably better characterized than lipid remodeling associated with tau accumulation, axonal degeneration, and tau-independent neuronal loss. Direct genotype-stratified evidence for APOE ε4-dependent cholesterol transport, astrocytic lipid-droplet formation, and cholesteryl-ester accumulation also remains limited. Similarly, lipid patterns observed in familial-AD-related transgenic models cannot yet be assumed to represent sporadic late-onset AD, in which aging, vascular pathology, metabolic disease, inflammatory history, and heterogeneous amyloid and tau burdens interact over decades [17,65,75,77]. These gaps define the central biological questions for future spatial lipidomic studies.

Analytically, MALDI-MSI has progressed from regional maps of nominal *m*/*z* values toward multidimensional molecular histology. MALDI-2 and other post-ionization strategies can increase sensitivity; ion mobility and structure-selective fragmentation can reduce isobaric and isomeric ambiguity; reactive matrices and derivatization can expand coverage to cholesterol, neutral lipids, fatty acids, and oxidized species; and spatial multiomics can connect lipid distributions with Aβ, tau, glial markers, neuronal structures, glycans, proteins, and tissue architecture [44,46,58,61,72,77,87,94,95,96,97,98,99,100,101,102,103,104,105,106,107,108,109,110,111,112,113,114,115,116,117,118].

However, additional analytical dimensions do not automatically produce more reliable biological conclusions. Post-ionization enhancement, mobility separation, derivatization yield, spatial registration, and computational annotation introduce their own forms of class-dependent and tissue-dependent variability.

Future studies should therefore be designed around specific unresolved biological questions rather than maximum technical complexity. The highest priorities are to determine when lipid remodeling first appears relative to amyloid and tau pathology; distinguish causal, compensatory, and secondary lipid changes; identify the cellular origins of plaque-associated lipid species; define the influence of APOE genotype and disease subtype; and establish which spatial signatures are preserved across models, human cohorts, brain regions, laboratories, and analytical platforms. Addressing these questions will require longitudinal experimental models, neuropathologically staged human tissue, APOE-stratified cohorts, cell-type-resolved markers, structural lipid identification, and anatomically harmonized sampling.

Translation will additionally depend on quantitative credibility and reproducibility. Ion intensity cannot be equated directly with lipid concentration, and mass-based annotation alone is insufficient when adducts, fragments, isobars, and structural isomers are present [41,119,120,121]. Harmonized tissue handling, matrix deposition, internal-standard strategies, orthogonal LC–MS/MS validation, transparent annotation levels, shared reference materials, patient-level replication, and multicenter validation are therefore prerequisites for distinguishing robust disease biology from laboratory-specific effects [122,123,124,125,126,133,134,135,136,137,138,139,140,141,142].

The most realistic near-term application of lipid MALDI-MSI is molecular neuropathology and therapeutic-response mapping rather than routine ante-mortem diagnosis. Its principal value lies in revealing molecularly distinct lesions, spatially restricted glial and myelin responses, region-specific lipid vulnerability, and treatment-associated biochemical changes that cannot be resolved by conventional homogenate lipidomics [127,128,129,130,131,143,144]. Under rigorous analytical and neuropathological validation, MALDI-MSI can evolve from a descriptive imaging platform into a mechanistically informative framework for defining how lipid metabolism contributes to the initiation, progression, and heterogeneity of Alzheimer’s disease [21,75,79,122,123,124,125,126,127,128,129,130,131,132,133,134,135,136,137,138,139,140,141,142,143,144].

## Figures and Tables

**Table 1:** Core analytical dimensions of lipid MALDI-MSI and their implications for data interpretation.

Analytical Dimension	Definition	Principal Determinants	Consequence for Lipid Analysis	Main Interpretative Risk
Ionization efficiency	Probability that an analyte forms a detectable ion	Matrix chemistry, polarity, laser fluence, tissue composition	Determines lipid-class coverage and signal intensity	Absence of a signal interpreted as absence of the lipid [10,19,20]
Ionization polarity	Charge mode used for molecular detection	Lipid headgroup, matrix acidity or basicity, adduct chemistry	Selects complementary lipid classes	One polarity interpreted as representative of the complete lipidome [10,19,22,23]
Spatial resolution	Minimum distance at which molecular distributions can be differentiated	Laser diameter, raster step, crystal size, analyte migration	Determines whether regional, plaque-level, or cellular structures can be resolved	Nominal pixel size equated with true independent resolution [17,21,24]
Sensitivity	Ability to detect low-abundance ions	Sampled area, ion yield, detector performance, post-ionization	Controls detection of minor and poorly ionizing lipids	High spatial resolution interpreted without considering signal loss [21,22,24]
Mass resolving power	Ability to separate ions with closely spaced *m*/*z* values	Mass analyzer and acquisition parameters	Reduces overlap of near-isobaric peaks	Unresolved species treated as one molecule [17,21]
Mass accuracy	Agreement between measured and theoretical *m*/*z*	Calibration, instrumental stability, ion abundance	Narrows possible elemental compositions	Accurate mass treated as definitive structural identification [17,21]
Structural specificity	Ability to distinguish isomers and molecular structures	MS/MS, ion mobility, derivatization, authentic standards	Supports assignments beyond lipid class and sum composition	Isomeric lipids reported as uniquely identified species [21,25]
Fragmentation control	Ability to distinguish endogenous ions from source-generated products	Laser fluence, matrix, source conditions, molecular stability	Prevents fragments from being classified as separate metabolites	In-source fragments interpreted as endogenous lipid species [26]
Quantitative reliability	Relationship between ion intensity and analyte abundance	Ion suppression, internal standards, normalization, matrix effects	Determines comparability among regions and specimens	Ion intensity interpreted directly as absolute concentration [20,21]

**Table 2:** Key preanalytical and matrix-deposition variables affecting lipid MALDI-MSI data quality.

Workflow Stage	Main Analytical Risk	Consequence for Lipid Imaging	Recommended Control
Tissue collection	Delay before freezing and variable post-mortem interval	Lipid hydrolysis, oxidation, and artifactual lysolipid formation	Standardize collection time and report post-mortem metadata [27,28,29]
Freezing and storage	Slow cooling, temperature fluctuations, and repeated freeze–thaw cycles	Tissue distortion, membrane disruption, and increased technical variability	Use one validated rapid-freezing method and stable low-temperature storage [27,28,29]
Cryosectioning	Inconsistent thickness, temperature, or anatomical depth	Variable analyte load and non-comparable regional profiles	Standardize section thickness, cryostat settings, and sampling level [27,29,30]
Embedding and mounting	OCT contamination, poor adhesion, or nonconductive surfaces	Background ions, ion suppression, and localized signal loss	Minimize OCT contact, use conductive slides, and include embedding blanks [27,31]
Drying and pretreatment	Residual moisture or nonselective washing	Analyte migration, selective lipid extraction, and altered adduct patterns	Apply controlled drying and validate washing on adjacent sections [27,32,33]
Matrix deposition	Excessive solvent exposure, coarse crystals, or uneven coating	Reduced spatial fidelity and heterogeneous ionization	Optimize spraying, sublimation, or dry deposition for the target resolution [34,35,36,37,38]
Matrix performance	Reliance on total peak number alone	Matrix clusters, fragments, and redundant adducts counted as lipid features	Evaluate lipid-class coverage, background, fragmentation, and image contrast [39]
Batch design and quality control	Disease group confounded with preparation or acquisition batch	False statistical separation and poor reproducibility	Randomize samples and include pooled reference sections and standards [20,40,41]
Quantification and validation	Ion intensity treated as absolute concentration	Overinterpretation of abundance differences	Use class-matched internal standards and orthogonal LC–MS/MS validation [40,41,42]
FFPE and multimodal workflows	Lipid extraction, reagent interference, and registration error	Residual lipid profiles or inaccurate pathological co-localization	Validate the complete workflow and interpret FFPE separately from fresh-frozen tissue [43,44,45,46]

**Table 4:** Principal barriers and recommended actions for translating lipid MALDI-MSI into Alzheimer’s disease research and clinical neuropathology.

Translational Barrier	Main Consequence	Recommended Action
Variable tissue collection and storage	Lipid degradation, oxidation, and batch effects	Harmonized post-mortem, freezing, storage, and metadata protocols [122,123,124]
Inconsistent sectioning and matrix deposition	Variable signal intensity and spatial resolution	Fixed section thickness, automated deposition, and predefined acceptance criteria [20,124]
Limited interlaboratory reproducibility	Site-specific lipid signatures	Multicenter ring trials, shared reference sections, and harmonized acquisition [125,141,142]
Incomplete quantification	Ion intensity misinterpreted as concentration	Multiclass internal standards, calibration models, and LC–MS/MS quality control [41,122,123]
Ambiguous lipid annotation	Incorrect biological pathway interpretation	MS/MS, ion mobility, reference spectra, confidence scoring, and standardized nomenclature [119,133,134,135,136,137,138,139,140]
Small and heterogeneous cohorts	Overfitting and poor generalizability	Patient-level replication, independent validation, and neuropathological stratification [75,77]
Complex computational workflows	Low transparency and limited reproducibility	Version-controlled pipelines, raw-data access, and SMART-compliant reporting [126,132,140]
Undefined clinical purpose	Inappropriate validation and regulatory uncertainty	Predefined intended use, performance thresholds, and clinical decision framework [127,130,131]

## Data Availability

No new data were created or analyzed in this study. Data sharing is not applicable to this article.

## Abbreviations

The following abbreviations are used in this manuscript:

9-AA	9-Aminoacridine
AD	Alzheimer’s disease
Aβ	Amyloid-β
APOE	Apolipoprotein E
APOE ε4	Apolipoprotein E ε4 allele
APP/PS1	Amyloid precursor protein/presenilin 1 transgenic model
APPPS1	Amyloid precursor protein/presenilin 1 transgenic model
ATT	6-Aza-2-thiothymine
CCS	Collision cross section
CE	Cholesteryl ester
CERAD	Consortium to Establish a Registry for Alzheimer’s Disease
CHCA	α-Cyano-4-hydroxycinnamic acid
DAN	1,5-Diaminonaphthalene
DBDA	DBDA matrix
DESI	Desorption electrospray ionization
DHA	Docosahexaenoic acid
DHAP	2,5-Dihydroxyacetophenone
DHB	2,5-Dihydroxybenzoic acid
FFPE	Formalin-fixed, paraffin-embedded
FT-ICR	Fourier transform ion cyclotron resonance
GM1	Monosialotetrahexosylganglioside
GM2	Monosialotrihexosylganglioside
GM3	Monosialodihexosylganglioside
ITO	Indium tin oxide
LC	Liquid chromatography
LC–MS	Liquid chromatography–mass spectrometry
LC–MS/MS	Liquid chromatography–tandem mass spectrometry
LPS	Lipopolysaccharide
MALDI	Matrix-assisted laser desorption/ionization
MALDI-2	MALDI followed by laser-based post-ionization
MALDI-MS	Matrix-assisted laser desorption/ionization mass spectrometry
MALDI-MSI	Matrix-assisted laser desorption/ionization mass spectrometry imaging
MALDI-TIMS	Matrix-assisted laser desorption/ionization–trapped ion mobility spectrometry
MALDI-TOF	Matrix-assisted laser desorption/ionization time-of-flight mass spectrometry
MRM-MS	Multiple-reaction-monitoring mass spectrometry
MS	Mass spectrometry
MS/MS	Tandem mass spectrometry
MS1	Full-scan or precursor-ion mass spectrometry
MSI	Mass spectrometry imaging
NEDC	N-(1-Naphthyl)ethylenediamine dihydrochloride
OCT	Optimal cutting temperature compound
OzID	Ozone-induced dissociation
PA	Phosphatidic acid
PC	Phosphatidylcholine
PE	Phosphatidylethanolamine
PG	Phosphatidylglycerol
PI	Phosphatidylinositol
PS	Phosphatidylserine
SM	Sphingomyelin
SMART	Data reporting standard for mass spectrometry imaging
TIMS	Trapped ion mobility spectrometry
TIMS-MSI	Trapped ion mobility spectrometry–mass spectrometry imaging
UHPLC	Ultra-high-performance liquid chromatography
UHPLC–MRM-MS	Ultra-high-performance liquid chromatography–multiple-reaction-monitoring mass spectrometry
